# Deconvoluting hepatic processing of carbon nanotubes

**DOI:** 10.1038/ncomms12343

**Published:** 2016-07-29

**Authors:** Simone Alidori, Robert L. Bowman, Dmitry Yarilin, Yevgeniy Romin, Afsar Barlas, J. Justin Mulvey, Sho Fujisawa, Ke Xu, Alessandro Ruggiero, Vladimir Riabov, Daniel L. J. Thorek, Hans David S. Ulmert, Elliott J. Brea, Katja Behling, Julia Kzhyshkowska, Katia Manova-Todorova, David A. Scheinberg, Michael R. McDevitt

**Affiliations:** 1 Department of Radiology, Memorial Sloan-Kettering Cancer Center, New York 10065, USA; 2 Department of Molecular Pharmacology and Chemistry, Memorial Sloan-Kettering Cancer Center, New York 10065, USA; 3 Molecular Cytology Core Facility, Memorial Sloan-Kettering Cancer Center, New York 10065, USA; 4 Department of Radiology, Papworth Hospital NHS Foundation Trust, Cambridge University Health Partners, Cambridge CB23 3RE, UK; 5 Institute of Transfusion Medicine and Immunology, Medical Faculty Mannheim, Ruprecht-Karls University of Heidelberg, Mannheim 68167, Germany; 6 Laboratory for Translational Cellular and Molecular Biomedicine, Tomsk State University, Tomsk 634050, Russia; 7 Department of Radiology and Radiological Sciences, Johns Hopkins School of Medicine, Baltimore, Maryland 21205, USA; 8 Red Cross Blood Service Baden-Württemberg-Hessen, Mannheim 68167, Germany; 9 Department of Pharmacology, Weill Cornell Medical College, New York 10065, USA; 10 Department of Medicine, Weill Cornell Medical College, New York 10065, USA

## Abstract

Single-wall carbon nanotubes present unique opportunities for drug delivery, but have not advanced into the clinic. Differential nanotube accretion and clearance from critical organs have been observed, but the mechanism not fully elucidated. The liver has a complex cellular composition that regulates a range of metabolic functions and coincidently accumulates most particulate drugs. Here we provide the unexpected details of hepatic processing of covalently functionalized nanotubes including receptor-mediated endocytosis, cellular trafficking and biliary elimination. Ammonium-functionalized fibrillar nanocarbon is found to preferentially localize in the fenestrated sinusoidal endothelium of the liver but not resident macrophages. Stabilin receptors mediate the endocytic clearance of nanotubes. Biocompatibility is evidenced by the absence of cell death and no immune cell infiltration. Towards clinical application of this platform, nanotubes were evaluated for the first time in non-human primates. The pharmacologic profile in cynomolgus monkeys is equivalent to what was reported in mice and suggests that nanotubes should behave similarly in humans.

## Abstract (web summary)

Application of carbon nanotubes as drug delivery carriers is stalled by uncertainties over their distribution and toxicity *in vivo*. Here, the authors use animal models to show that, while the bulk of nanotubes is renally cleared, a fraction can be eliminated through an alternative hepatobiliary pathway.

## Graphical abstract

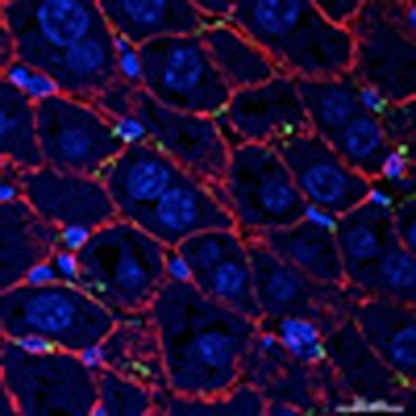

Single-wall carbon nanotubes (SWCNT) have attracted immense interest as a platform for pharmaceutical interventions due to their unique physicochemical properties and remarkable fibrillar pharmacology^1^,^2^. These high aspect ratio macromolecules can be readily derivatized via covalent chemical or non-covalent physical means, which advocates for their consideration in the development of multi-functional scaffolds capable of targeting disease for diagnostic imaging or therapeutic drug delivery^1^,^3^,^4^. Despite the potential to assume a novel position in medical applications, carbon nanotubes have not entered the clinic due to a limited understanding of their fate *in vivo* as well as a lack of consideration for the role that functionalization confers to pharmacology^3^. Sidewall-functionalized SWCNT, covalently appended with multiple primary amines^5^, have been shown to undergo very rapid renal elimination in animal models via glomerular filtration^6^,^7^,^8^,^9^,^10^,^11^,^12^. Thus, these water-soluble macromolecules exhibited a pharmacokinetic profile similar to that of a small molecule one-thousandth their size because they possess a high aspect ratio^6^,^8^,^11^.

Most particulate drugs are scavenged and processed by the liver^1^. Hepatic accumulation and subsequent hepatobiliary elimination of functionalized nanotubes has been reported in animal models, however, the fraction of the administered dose that localized in the liver varied tremendously in correlation with the methods used to functionalize the nanotube^6^,^7^,^8^,^9^,^10^,^11^,^12^,^13^,^14^,^15^,^16^,^17^,^18^,^19^,^20^,^21^,^22^,^23^,^24^. Furthermore, the hepatic location of SWCNT has generally been described as liver accumulation with only limited identification of the cell populations involved in accretion and no information on the processes of receptor-mediated endocytosis^25^,^26^ or explanation of hepatobiliary elimination. The liver is an essential organ with a complex cellular architecture that regulates an extensive range of metabolic functions. The liver is comprised primarily of hepatocytes, Kupffer cells, liver sinusoidal endothelium (LSEC), stellate cells, bile duct epithelium and vascular endothelium. Mapping the cytotopographic distribution and hepatic processing of functionalized nanotubes is required for understanding their fate *in vivo*. These data will, in turn, inform on the optimal applications and possible toxicities of these unique materials with wider import for nanomedicine as a whole.

This paper reports several novel and unexpected details of hepatic processing of covalently functionalized nanotubes including receptor-mediated endocytosis, cellular trafficking and biliary elimination. First, water-soluble ammonium-functionalized fibrillar nanocarbon is found to preferentially localize in the fenestrated sinusoidal endothelium of the liver but not hepatocytes or resident macrophages. Second, both scavenger Stabilin receptors mediate the endocytic clearance of nanotubes. This particular receptor specificity for nanocarbon leads us to find that lymph tissue also accumulates carbon nanotubes. Third, we propose a mechanism for the hepatobiliary elimination of nanocarbon. Fourth, biocompatibility is evidenced by the absence of cell death and no immune cell infiltration. Towards clinical application of this platform, nanotubes were evaluated for the first time in non-human primates. The pharmacologic profile in cynomolgus monkeys is equivalent to what was reported in mice and suggests that nanotubes should behave similarly in humans.

## Results

### Nanomaterial synthesis and characterization

**Figure 1:**
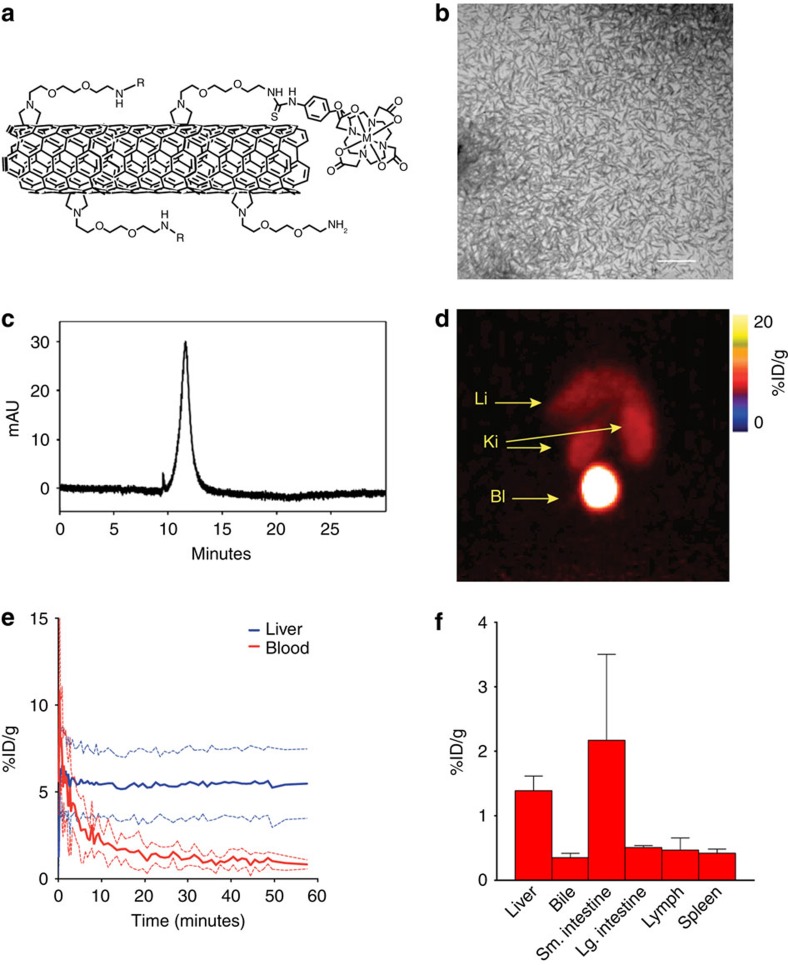
Nanomaterial characterization and murine pharmacokinetics. (**a**) Graphic illustration of the ammonium-functionalized single walled carbon nanotube (fCNT) appended with a DOTA radio-metal ion chelate. NB, Figures are not drawn to scale. (**b**) TEM image of purified fCNT (scale bar, 500 nm). (**c**) Reverse-phase HPLC chromatograph of fCNT monitoring the signature ultraviolet–visible absorbance at 330 nm. (**d**) PET projection image of a representative mouse at 60 min post injection. The notations Ki, Li, and Bl indicate the kidneys, liver and bladder, respectively. The head is positioned at the top of the image. (**e**) Time–activity curves generated from region-of-interest analysis (%ID g^−1^ (mean±s.d.)) of liver accumulation (blue lines) and blood compartment clearance (red lines) in four mice that were PET imaged. (**f**) Biodistribution data for select tissue and bile (%ID g^−1^ (mean±s.e.m.)) 1 h after administration. (NB, A comprehensive pharmacokinetic data set is shown in the Supplementary Fig. 2.)

An ammonium-functionalized carbon nanotube (fCNT) was used to probe hepatic processing (Fig. 1a). This nanomaterial was prepared and characterized as previously described^8^. Transmission electron microscopy analysis of solid fCNT confirmed a mean length of 195±69 nm (Fig. 1b). These fCNT were water soluble (10 g l^−1^) and chromatographically characterized (Fig. 1c); in addition, they were rapidly renally filtered in both murine^6^,^8^,^11^ and primate models. The Raman radial breathing mode feature (234 cm^−1^) yielded a nanotube diameter of 0.96 nm (ref. 6). These amino-functionalized nanotubes were further appended with fluorescent moieties and metal ion chelates to report location via multiple imaging modalities and investigate whole body, organ, cell and intracellular distribution.

### Murine pharmacokinetic profile of fCNT

Intravenously administered fCNT rapidly cleared the blood and a fraction accreted in the liver or was eliminated intact via the bile. Dynamic positron emission tomographic (PET) imaging and tissue collection were performed to assess the whole body pharmacokinetic profile in a naive animal model. High contrast images of a representative mouse (Fig. 1d) showed that the activity cleared the vascular compartment by 1 h and was predominantly accreted in liver, spleen and kidney (earlier images at 3 and 20 min are shown in Supplementary Fig. 1). The balance of the injected dose (80–85%) was rapidly eliminated to the urine^8^ as shown by the bladder image at 60 min (Fig. 1d). Time–activity curves for blood and liver graphically show rapid clearance from the blood (Fig. 1e) and swift accumulation of a fraction of the injected dose (ID) in the liver (Fig. 1e,f). Detailed tissue harvest experiments (Fig. 1f and Supplementary Fig. 2) measured the %ID that partitioned into the blood, tissue, and bile as a function of time. Activity was measured in the liver (1.39±0.39%ID g^−1^), bile (0.35±0.15%ID g^−1^), spleen (0.42±0.11%ID g^−1^), lymph node (0.47±0.42%ID g^−1^) and small intestines (the duodenum, jejunum, and ileum contained 2.17±2.31%ID g^−1^) at 1 h. Radiochromatographic analysis of bile harvested from the gall bladder indicated intact fCNT (Supplementary Fig. 3).

### LSEC accumulation of fCNT

**Figure 2:**
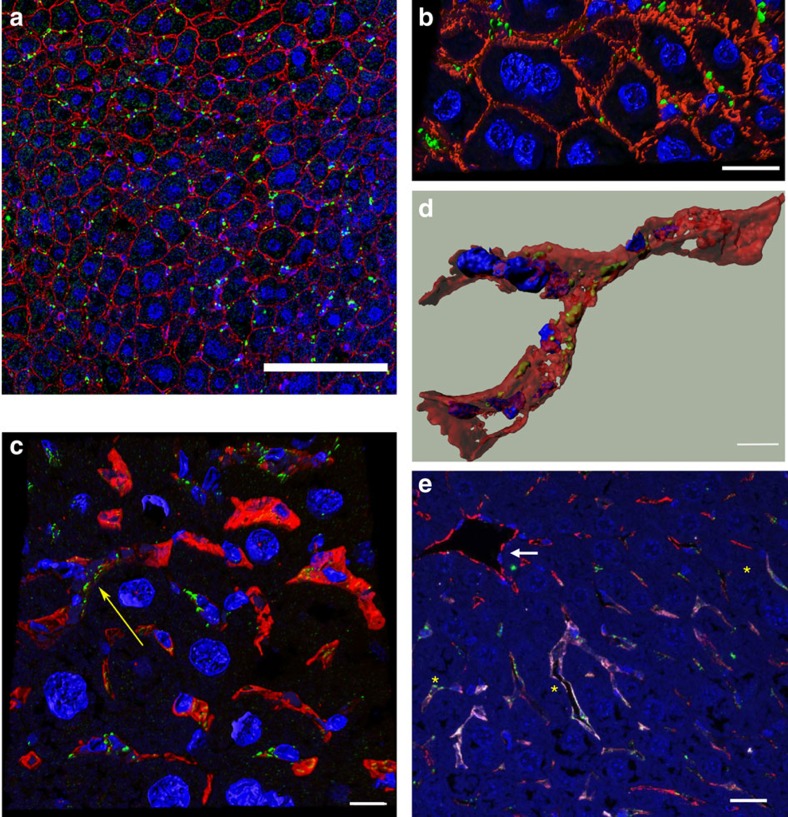
Murine liver sinusoidal endothelium accumulated fCNT *in vivo*. (**a**) fCNT was identified in the hepatic sinusoids at 1 h using anti-AF488 (green) to map AF488 that was covalently appended to fCNT; N-cadherin (red) was used to demarcate hepatocyte plasma membrane, and 4′, 6-diamidino-2-phenylindole (DAPI) (blue) to mark nuclei (scale bar, 25 μm). (**b**) A three-dimensional (3D)-rendered image of a confocal z-stack of the sinusoid at 1 h demonstrated that the anti-AF488 (green) stain was localized within the sinusoidal spaces and associated with smaller nucleated cells but not with any of the large nucleated hepatocytes. Tissue was stained with anti-AF488 (green)/DAPI (blue)/N-cadherin (red) (scale bar, 25 μm). (**c**) A 3D-rendered image of a confocal z-stack of the sinusoid shows LSEC marked with Lyve1 (red), nuclei with DAPI (blue), and fCNT with anti-AF488 (green) (scale bar, 10 μm). The yellow arrow shows LSEC with Lyve1 marker circumscribed around the fCNT signal (This cell is abstracted from the image and shown in **d**). (**d**) A 3D-rendered image of the LSEC (identified in Fig. 2c with the yellow arrow) revealed that the Lyve1 (red) marker entirely circumscribed the anti-AF488 (green) signal (scale bar, 4 μm). (**e**) The liver VE did not accumulate fCNT (white solid arrow) while the fenestrated LSEC did accumulate fCNT (yellow asterisks). The liver vascular endothelium was stained with CD31 (red), LSEC marked with Lyve1 (white) and CD31, fCNT (appended with AF488) were located with anti-AF488 stain (green) and nuclei with DAPI (blue) (scale bar, 10 μm).

Liver accumulated fCNT was localized exclusively in the hepatic sinusoids, associated with small-nucleated cells lining the sinusoids (Fig. 2, Supplementary Figs 4 and 5). fCNT was imaged using anti-AF488 immunofluorescence (IF) staining that was directed at AF488 moieties covalently appended onto the nanotube sidewall. This anti-AF488 probe was multiplexed with an array of co-stains selected to distinguish distinct cell types and organelles. The absence of fCNT in hepatocytes was corroborated with N-cadherin staining that delineates the hepatocyte plasma membrane. Controls included mice that were injected with AF488-alone (Supplementary Fig. 4) or vehicle (Supplementary Fig. 5) and both were negative for AF488 signal.

The fenestrated LSE that lines the hepatic sinusoids was the structure that localized fCNT as confirmed by multiplex IF detection using CD31, Lyve1 (lymphatic vessel endothelial hyaluronan receptor), anti-AF488, and DAPI stained liver sections (Fig. 2c–e, Supplementary Fig. 6). CD31 is a pan-endothelial marker while Lyve1 is a marker for lymphatic vascular endothelial cells; LSEC express Lyve1 and are a notable exception. The CD31 and Lyve1 membrane markers entirely circumscribed the anti-AF488 signal, substantiating fCNT clearance into the LSEC population (Fig. 2d). Vascular endothelium (VE) within the liver did not accumulate fCNT (Fig. 2e) and called attention to the differential capacities of specialized fenestrated LSEC and VE to accumulate and internalize fCNT. Liver sections stained with MECA32, another VE cell marker, confirmed this finding. Resident Kupffer cells (Supplementary Fig. 7) and bile duct epithelium (Supplementary Fig. 8) did not accumulate fCNT.

### Receptor-mediated endocytosis and organelle trafficking

**Figure 3:**
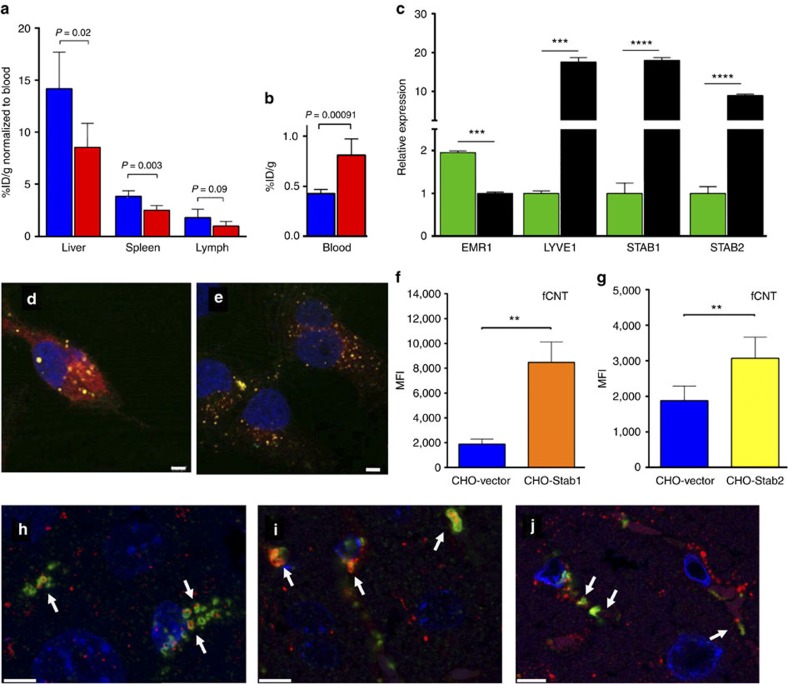
Stabilin-mediated endocytosis of fCNT and LSEC trafficking. (**a**) Mice were administered 20 mg heparin (blue bars) and 0.01 mg fCNT showed decreased fCNT accumulation in liver, spleen and lymph nodes versus heparin null controls (red bars) and (**b**) concomitantly more fCNT remained in the blood of the heparin treated mice after 1 h. (**c**) PCR analysis of LSEC (black bars) and Kupffer cells (green bars) isolated from mouse liver showed that the LSEC strongly expressed mRNA for both Stabilin-1 and Stabilin-2. The phenotype of each cell type was confirmed by FACS analysis (Supplementary Fig. 10). Immunofluorescent confocal images of CHO cells transfected with full-length human (**d**) Stabilin-1 (scale bar, 2.80 μm) or (**e**) Stabilin-2 (scale bar, 4.38 μm) expressing cells were stained with anti-Stabilin-1 and anti-Stabilin-2 antibodies (red) and anti-AF488 (green) to mark fCNT appended with AF488, and DRAQ5 to mark nuclei (blue). Punctate cellular structures with co-localized staining were evident in both cases. FACS analysis quantified bound and internalized fCNT in the CHO-transfected (**f**) Stabilin-1 (orange bar) and (**g**) Stabilin-2 (yellow bar) cells versus CHO transfected with an empty vector (blue bar, negative control). The bars are mean±SD using data from 6 experiments (**P<0.01). acLDL served as a positive control (Supplementary Fig. 13). Organelle trafficking of intracellular fCNT (anti-AF488 (green)) was observed within LSEC in mouse tissue in punctate patterns and was shown through co-staining to be associated with different intracellular organelles as a function of time. 3D-rendered confocal images of the (**h**) early endosome (EEA-1, red) at 5 min after injection; (**i**) Golgi compartments (Giantin, red) at 40 min; and (**j**) lysosomes (LAMP1, red) at 1 day. A complete time course of these representative data is shown in Supplementary Fig. 14. White arrows indicate co-localized AF488 signal with respective organelles. GM130 stained tissue data correlated with the Giantin stained sections and LAMP2 lysosome marker data (not shown) correlated with LAMP1. The nuclei are stained with DAPI (blue) and scale bars, 5 μm.

Stabilin-1 and Stabilin-2 are scavenger receptors expressed by LSEC and both mediated internalization of fCNT based on our data *in vivo* and *in vitro*. Competitive inhibition of these two fasciclin-like hyaluronan receptor homologues with heparin reduced the accumulation of fCNT in liver, spleen and lymph node. Heparin has been reported to be a substrate for Stabilin-1 and Stabilin-2 (refs 27, 28, 29) and both receptors are expressed on the specialized sinusoidal endothelium of liver, spleen and lymph^29^,^30^,^31^. Heparin competition *in vivo* resulted in a decrease in liver, spleen and lymph clearance of fCNT versus heparin null controls (*P*=0.017, 0.0025 and 0.09, respectively) with concomitantly more fCNT in the blood compartment of the heparin treated mice (*P*=0.00091) as shown in Fig. 3a,b, respectively. LSEC isolated from livers strongly expressed mRNA for both Stabilins and in contrast to Kupffer cell expression (Fig. 3c and Supplementary Fig. 9). Isolated cell phenotype was confirmed by FACS analysis (Supplementary Fig. 10). Receptor specificity for fCNT was confirmed *in vitro* using CHO cells stably transfected with full-length human Stabilin-1 or Stabilin-2 expression constructs. Confocal microscopy showed punctate cellular structures in which fCNT (AF488-positive) co-stained with each anti-Stabilin-1 and anti-Stabilin-2 antibody, respectively (Fig. 3d,e; control images are shown in Supplementary Figs 11 and 12). Bound or internalized fCNT was quantified using FACS that showed significant clearance (Fig. 3f,g) versus CHO-transfected with an empty vector (negative control); acLDL binding served as positive control, (Supplementary Fig. 13a,b). Furthermore, the binding of fCNT was significantly reduced in these Stabilin-expressing CHO cells in the presence of a challenge from excess heparin *in vitro* (Supplementary Fig. 13c,d). Organelle trafficking of fCNT in the mouse liver was monitored as a function of time. Punctate anti-AF488 staining patterns were associated with EEA-1 stained early endosomes at 5 min after injection; with both GM130 and Giantin stained Golgi compartments at 5 min through 1 day; and Lamp1 stained lysosomes at 40 min and 1 day (Fig. 3h–j and Supplementary Fig. 14).

### Hepatic biocompatibility of fCNT

**Figure 4:**
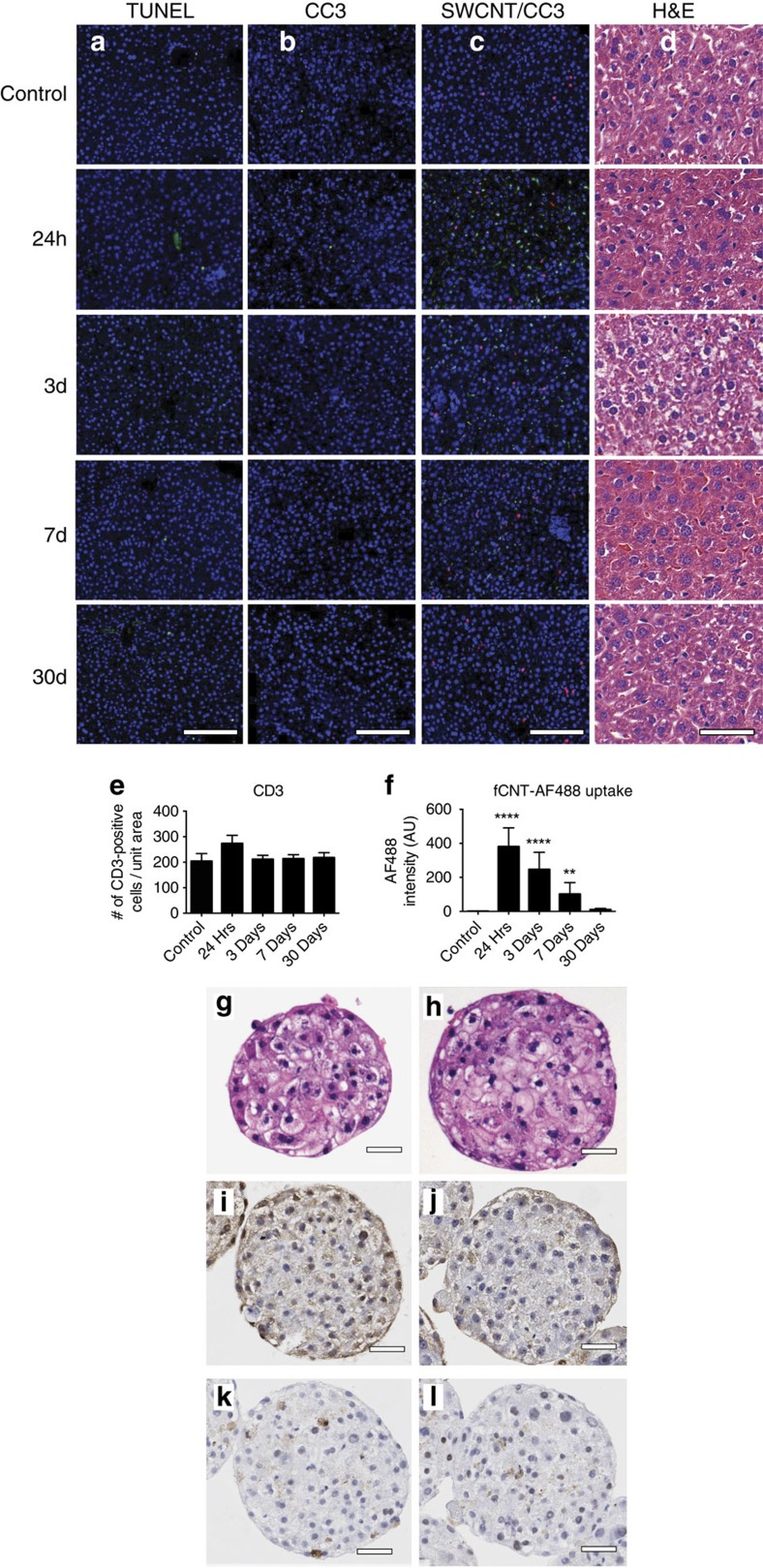
Hepatic biocompatibility of fCNT. The biocompatibility of fCNT in immunocompetent mice was examined as a function of time (1, 3, 7 or 30 days post injection) using (**a**) TUNEL and (**b**) cleaved caspase-3 (CC3) IF staining (green) of liver tissue versus untreated controls (scale bars, 150 μm for all panels). Few DNA fragmentation or apoptotic events were noted in the fCNT-treated animals relative to untreated control mouse livers. (**c**) T cell infiltration was imaged using CD3 staining (red). fCNT appended with AF488 was stained green with anti-AF488. (**d**) H&E stained mouse tissues corresponding to (**a**–**c**) above. Scale bars are 200 μm for all panels. (**e**) The normalized number of CD3-positive T cells remained stable throughout the time course of the study (*P*>0.01 compared with untreated control for all time points) and was not different compared with untreated control. (**f**) The green fCNT signal decreased over 30 days (****P<0.0001; **P<0.01, Student's *t*-test compared with the untreated control tissue. Images of human hepatocyte microspheres exposed to 30 mg l^−1^ fCNT for 1 day *in vitro* remained viable. No morphological or apoptotic differences were observed by H&E (**g**,**h**), TUNEL (**i**,**j**) or cleaved caspase-3 (**k**,**l**) staining of untreated control hepatocyte microspheres versus fCNT-treated hepatocyte microspheres, respectively. Scale bar, 50 μm in all images. The complete 3 day treatment dataset and positive control tissues are shown in Supplementary Fig. 15.

Biocompatibility of functionalized nanotubes in mice was examined as a function of time (1, 3, 7 or 30 days post injection) using terminal deoxynucleotidyl transferase dUTP nick end labelling (TUNEL) and cleaved caspase-3 (CC3) IF staining of immunocompetent mouse liver versus untreated controls. Few DNA fragmentation (Fig. 4a) or apoptotic (Fig. 4b) events were noted in either the fCNT-treated or untreated control mouse livers. Further, no T cell infiltration was detected by CD3 staining (Fig. 4c). No morphological differences between treated and control mouse liver was observed by hematoxylin and eosin (H&E) staining (Fig. 4d). The normalized number of CD3-positive T cells remained stable throughout the time course of the study (*P*>0.01 compared with untreated control for all time points) and was not different compared to untreated control (Fig. 4e). The AF488-fCNT signal decreased with time (Fig. 4f). Human hepatocyte microspheres were exposed to fCNT (15 and 30 mg l^−1^) for 3 days *in vitro* and remained viable at concentrations exceeding those used *in vivo*. No morphological or apoptotic differences between treated and control spheres was observed by H&E, TUNEL or CC3 staining (Fig. 4g–l and Supplementary Fig. 15).

### Pharmacokinetics and toxicology of fCNT in non-human primates

**Figure 5:**
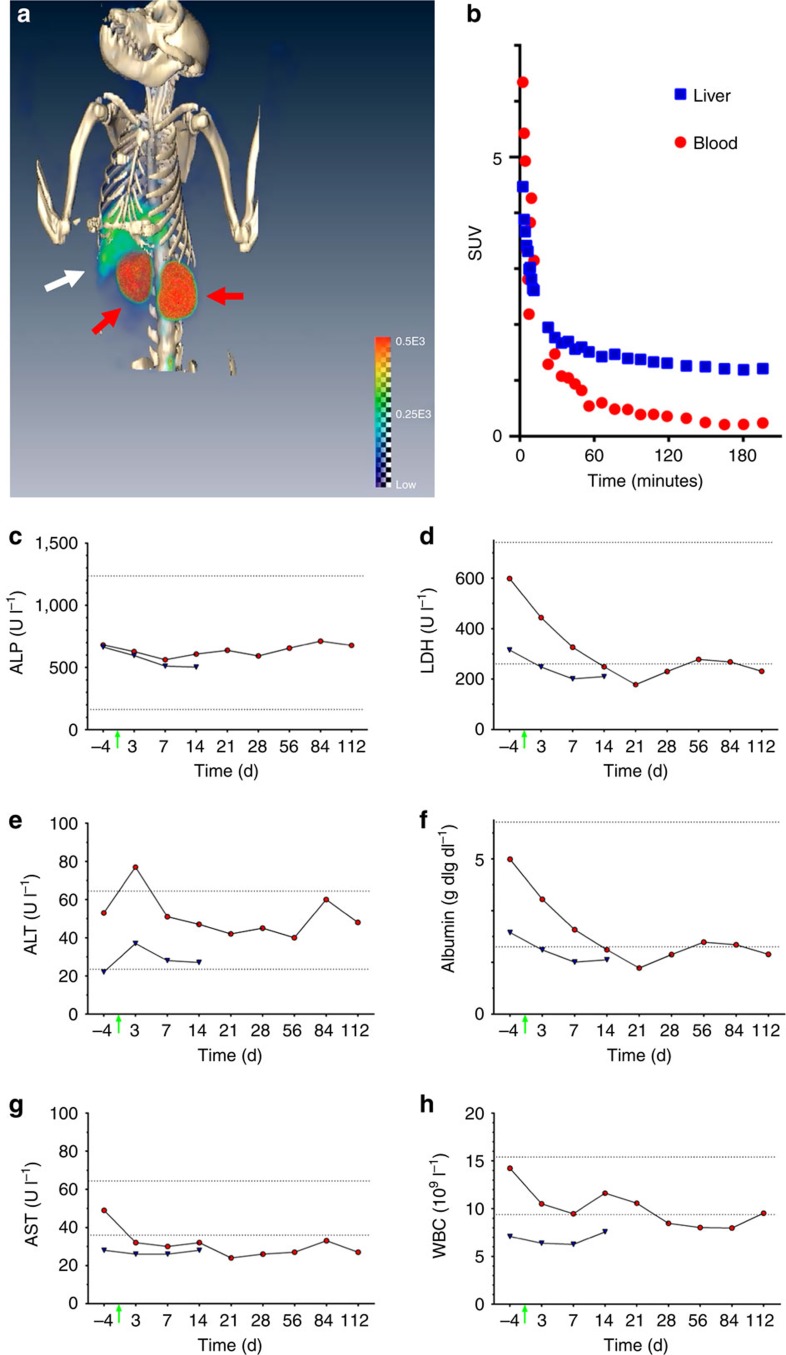
Pharmacokinetics and toxicology of fCNT in non-human primates. A pair of 5 kg cynomolgus monkeys received an intravenously administered dose of [^86^Y]fCNT (1 mg kg^−1^). (**a**) A fused PET/CT image of [^86^Y]fCNT showing liver (white arrow) and kidney (red arrows) distribution. (**b**) Time–activity curves for blood (red circles) washout and hepatic (blue squares) uptake. Biomarkers for hepatic functions in the pair of animals (red circles and blue triangles) were unchanged relative to baseline values at day minus 4 (NB, the green arrow indicates the time of injection and imaging). The values for (**c**) alkaline phosphatase (ALP), (**d**) lactate dehydrogenase (LDH), (**e**) alanine aminotransferase (ALT), (**f**) albumin, (**g**) aspartate aminotransferase (AST), and (**h**) white blood cell (WBC) count agreed with published ranges (dotted lines) for similarly aged male animals^32^.

Towards clinical application of this fCNT platform, the fCNT pharmacokinetic and toxicologic profiles were evaluated in naive non-human primates using PET-computed tomography (PET/CT) imaging, blood chemistry, haematological analyses and histopathology. The fibrillar nanomaterial exhibited similar blood clearance kinetics, tissue biodistribution, and renal elimination in a pair of 5 kg cynomolgus monkeys (*Macaca fascicularis*) as compared to mice. A 1 mg kg^−1^ dose of [^86^Y]fCNT was administered intravenously and had a blood half-life of 6–7 min. The majority of the dose was rapidly eliminated in the urine with a fraction accumulated in the liver and kidneys (SUV was 1 and 16, respectively). Figure 5a is a fused PET/CT image of [^86^Y]fCNT in a non-human primate showing liver and kidney distribution. Time–activity curves for blood washout and hepatic clearance were equivalent to what was observed in mice (Fig. 5b). The nanotubes were also biocompatible as shown by acute and chronic liver toxicity evaluations of blood chemistry and haematological indices. Biomarkers indicative of hepatic injury (alkaline phosphatase, lactate dehydrogenase, alanine aminotransferase, aspartate aminotransferase and albumin) and WBC counts were changed little relative to baseline values (Fig. 5c–h) and agreed with published ranges for similarly aged male animals^32^. Histopathological examination of liver, spleen, and lymph tissue (and other tissue) at necropsy by a pathologist was scored normal. These pharmacokinetic and toxicological fCNT profiles in primates suggest that the favourable pharmacological properties of fCNT should translate in humans.

## Discussion

Nanoparticles, in general, are severely limited by untoward hepatic clearance and lack of renal clearance. While the bulk of fCNT are renally cleared^6^,^7^,^8^,^9^,^10^,^11^, the next most prominent organ contributing to their accumulation and elimination is the liver. Data reported herein show the unexpected result that liver processed fCNT either by receptor-mediated LSEC scavenging or hepatobiliary elimination. Hepatic biocompatibity of these nanomaterials was confirmed in rodents and primates and is now explained by a combination of specific LSEC scavenging and intact biliary clearance. Hepatic and renal processing and clearance of this fibrillar nanomaterial are advantageous biological properties.

Given the important role of the liver in mammalian physiology and homoeostasis, the absence of fCNT in parenchyma was an interesting and potentially significant finding that correlated with the observed biocompatibility of these covalently functionalized nanomaterials^6^,^7^,^8^,^9^,^10^,^11^,^12^,^13^,^14^,^15^,^16^,^33^,^34^,^35^. The various nonparenchymal cells that populate the liver sinusoid are interleaved and difficult to distinguish morphologically with light microscopic examination. However, in this study we employed specific multiple IF stains to identify and differentiate between Kupffer cells, LSEC^30^,^36^,^37^,^38^,^39^,^40^, and Stellate cells^41^. Our data demonstrate that soluble, molecular fCNT are scavenged by LSEC via a Stabilin receptor-mediated process. Our physicochemical characterizations indicated that fCNT is soluble and well dispersed in physiologic milieu and behaved like a fibrillar macromolecule rather than particulates which would be expected to be phagocytosed by Kupffer cells^38^. Accordingly, we observed no evidence of Kupffer clearance of fCNT using discrete cell markers to interrogate and unequivocally identify each sinusoidal cell phenotype. Furthermore, we confirmed that Kupffer cells did not express either Stabilin receptor which explained the lack of fCNT clearance by this resident macrophage.

The hypothesis that macromolecular fCNT remain soluble and individualized *in vivo* was supported by this study and previous reports^6^,^7^,^8^,^11^. In direct contrast, non-covalently modified SWCNT (for example, dispersed with surfactant or polyethylene glycol (PEG)) exist only for a brief interval in the blood before displacement of the solubilizing agent by serum protein^17^. This disadvantage of non-covalent dispersal is an existing methodological problem that makes our covalently functionalized CNT study relevant and impactful moving forward. Surfactant- and polyethylene glycol-dispersed nanocarbon suspensions are inherently unstable and readily aggregate *in vivo*, rendering these non-soluble materials susceptible to macrophage opsonophagocytosis^21^. In addition, since surfactant-dispersed nanomaterials were unable to efficiently clear renally, a greater majority of the injected dose accumulates in liver presumably in macrophages^17^,^18^,^19^,^20^,^21^,^22^. The untoward behaviour of non-covalently modified nanotubes *in vivo* is in direct contrast to soluble covalently functionalized fCNT that are rapidly filtered by the kidneys and exhibit only minimal accumulation in liver, spleen and lymph^6^,^7^,^8^,^9^,^10^,^11^,^12^,^13^.

The vascular endothelial termini in the liver, spleen and marrow are tortuous sinusoids. The hepatic sinusoids interface between the blood supply and hepatocytes and mediate scavenging and transport of blood-borne solutes. LSEC are fenestrated endothelium possessing numerous open fenestrae, without diaphragm or basement membrane^30^,^36^,^37^,^38^,^39^,^40^. A sieve plate morphology and high endocytic capacity support the unique role of LSEC in solute trafficking and active scavenging of a range of macromolecules and colloids that escape Kupffer phagocytosis. Punctate anti-AF488 staining of LSEC organelles indicated fCNT trafficking through the early endosome, Golgi, and lysosome compartments and supported receptor-mediated endocytosis as the mechanism of clearance^25^,^26^. Furthermore, we now provide evidence that Stabilin-1 and Stabilin-2 mediated endocytic clearance of fCNT *in vivo*. The Stabilins are scavenger receptors that recognize and clear a range of molecular substrates (for example, hyaluronan, dermatan sulfate and acetylated low density lipoprotein)^38^. Heparin clearance remains controversial notwithstanding strong evidence for Stabilin-1 and Stabilin -2 binding^27^,^28^,^29^,^30^,^31^ countered by reports that heparin is not a ligand for Stabilin-2 (refs 42, 43). Our new data definitely suggest that heparin did compete with fCNT clearance by LSEC *in vivo* and with both Stabilin-1- and Stabilin-2-expressing CHO cells *in vitro*. Heparin also reduced the bidodistribution of fCNT in spleen and lymph tissue.

Stellate cells reside in the hepatic perisinusoidal space of Disse intimately positioned between LSEC and hepatocytes^41^. The paracrine secretion of vascular endothelial growth factor by stellate cells and hepatocytes sustains the LSEC population and promotes autocrine production of nitric oxide by LSEC^30^. It has been shown that LSEC play an important role in maintenance of stellate cell quiescence and prevent their activation and loss of the vascular endothelial growth factor paracrine effect^44^. While LSEC were the predominant target for fCNT, there was rare evidence of fCNT in stellate cells (Supplementary Fig. 16). Because one function of the LSEC is to guard these cells and prevent activation^30^,^44^, occasionally fCNT may have been taken-up by stellate cells. It is not clear how or if the stellate cell recognized fCNT, but the literature on these perisinusoidal cells is still developing.

The vascular endothelial architecture serves as the primary conduit to distribute fCNT *in vivo* but there was no evidence that these cells accumulated this nanomaterial (Fig. 2e). This particular result calls attention to the differential functionality and receptor expression of these two endothelial cell types with continuous or discontinuous fenestrated cytoplasm. Cultured endothelial cells were observed to accumulate SWCNT, albeit under non-physiologic conditions over a prolonged time^45^, however, our PET imaging and IF studies have not identified any fCNT accumulation in the VE^6^,^8^,^11^.

Intact fCNT cleared the liver via secreted bile (Fig. 1f and Supplementary Figs 2 and 3). The presence of intact radiolabeled fCNT in the bile, intestines and faeces strongly supported a mechanism of hepatobiliary clearance. The epithelial bile duct cells that comprise these channels did not show any fCNT accretion (Supplementary Fig. 8). We propose that a portion of the fCNT in the sinusoid, not endocytosed by LSEC, diffused from the Disse space into the biliary canaliculi and subsequently mixed with bile. Because we observed no evidence of fCNT in hepatocytes and the bile contained intact nanomaterial, it is difficult to reconcile a mechanism whereby hepatocytes mediated transport of fCNT from blood to bile. Evidence does exist for a permeable barrier permitting pigments and cellular debris to bypass hepatocyte processing and transit directly from blood to bile^46^; scanning electron microscopy has shown 100 nm zones between the space of Disse and the bile canaliculi that were interpreted as sites for molecular diffusion^47^. These permeable Disse/canalicular junctions have been utilized to effect retrograde, non-viral gene therapy to the liver via infusion from the biliary tree^48^. Conventional views of hepatocyte-mediated elimination of blood-borne solutes into bile have overlooked this interesting diffusion process across the Disse/canalicular junction^49^.

The spleen and lymph nodes also accumulated fCNT and clearance was connected to the expression of both Stabilin receptors in these tissues^28^,^29^,^30^,^31^. The implications are exciting because one can now predict fCNT accumulation based on receptor recognition. Lymph node clearance of fCNT has not been reported until now and only discovered on the basis of lymph expression of Stabilin receptors. Based on our identification of Stabilin-mediated endocytosis of fCNT, we looked for and confirmed accumulation in lymph nodes. The specific accumulation of fCNT by these organs was observed in biodistribution studies (Fig. 1f and Supplementary Fig. 2) and blocked in heparin competition studies (Fig. 3a). In parallel with the liver, the specialized splenic sinusoidal endothelium (SSEC) also internalized fCNT while splenic macrophage clearance was not observed (Supplementary Fig. 17). The splenic sinusoids are tortuous VE termini lined with SSEC. The SSEC differ from LSEC in that they exhibit continuous cytoplasm and disorganized basement membrane^30^. The spleen is another important reticuloendothelial tissue and it paralleled the liver in the cell types that localized fCNT. Lymphatic endothelial cell accumulation of fCNT now warrants further investigation.

These data contribute significantly to our understanding of the hepatic processing of fCNT *in vivo*. The pharmacokinetic and safety profile of fCNT in a non-human primate strongly proposes extrapolation to humans. The predominant hepatic cell type that accumulated this fibrillar nanocarbon was a professional scavenger endothelial cell in the sinusoid that performed rapidly and at high capacity. Mouse LSEC has 14±5 fenestrae per μm^2^ (humans have 15–25 per μm^2^) with diameters of 99±18 nm (humans, 50<*d*<300 nm)^36^. This is an avid, dedicated mammalian scavenger cell and in combination with intact biliary elimination of fCNT has yielded a very favourable biological outcome for soluble fibrillar nanocarbon in animal models. Biocompatibility has been confirmed in several model systems and now these results explain the action of the host on fCNT and add further weight to arguments for use in man.

Potential therapeutic application of fCNT in humans could focus on the critical role of LSE in numerous liver disorders (for example, ischemia-reperfusion injury, sinusoidal obstructive syndrome, nonalcoholic steatohepatitis, and pseudocapillarization in the aging population)^30^. fCNT could be envisioned as a molecular platform to specifically target the LSEC and deliver therapeutic interventions (for example, small molecule drugs or RNA interference) to treat LSEC-based disorders. Hepato-targeted gene therapy strategies are being explored^50^,^51^ and fCNT could be included as a selective delivery platform based on our favourable pharmacokinetic and toxicologic data and the ability of fCNT to strongly bind, deliver and unload siRNA *in vivo*^52^,^53^. Macrophages that express Stabilin-1 (not liver-resident Kupffer cells) should accumulate fCNT via receptor-mediated processes and suggests further investigation^54^. Also, now that lymph node clearance of fCNT has been described, applications for lymph targeting strategies could be explored.

## Methods

### Synthesis and characterization of fCNTs

HiPCO SWCNT (Unidym) were covalently functionalized with primary amines, Alexa Fluor 488 tetrafluorophenyl ester (AF488-TFP, Invitrogen), AF680-SE (Invitrogen), and 2-(4-isothiocyanatobenzyl)-1, 4, 7, 10-tetraazacyclododecane-1, 4, 7, 10-tetraacetic acid (DOTA, Macrocyclics) and purified as described^8^. The amine loading per gram of SWCNT was determined using the Sarin assay as described previously^6^. Characterization was performed as described previously^6^,^8^,^14^ and confirmed the identity, stoichiometry and purity of the nanomaterial. Radiolabeling with ^86^Y and ^111^In for imaging and biodistribution was performed as previously described^6^,^8^,^14^.

### Dynamic PET imaging to investigate pharmacokinetics

Dynamic imaging was performed with the microPET Focus 120 (CTI Molecular Imaging) for the mouse model (♂, NCr/nu/nu, Taconic) as described^8^. Dynamic PET/CT imaging was performed with a Siemens Biograph mCT PET/CT system for the non-human primate model (♂, *Macaca fascicularis*, Charles River) and described in the Supplementary Information. For all *in vivo* experiments, housing and care were in accordance with the Animal Welfare Act and the Guide for the Care and Use of Laboratory Animals. The animal protocols were approved by the Institutional Animal Care and Use Committee at MSKCC.

### Biodistribution and elimination

Mice (♂, NCr/nu/nu, Taconic) received an IV injection of [^111^In]fCNT containing 0.04 mg of SWCNT construct and 74 kBq (0.002 mCi) of ^111^In per mouse via the retroorbital sinus. The animals were placed into 4 groups of 3–5 mice per group. Each group was sacrificed with CO_2_ aspiration at 1, 3, 24 h and 7 days. Tissue samples (blood, heart, kidneys, muscle, bone, lung, stomach, liver, spleen, lymph nodes (superficial cervical, axillary, brachial, renal inguinal and lumbar), bile, small intestine (consisting of the duodenum, jejunum and ileum), contents of the small intestine, large intestine (consisting of the caecum and colon), contents of the large intestine), and faeces were collected, weighed and counted using a γ-counter (Packard Instrument, Co.) with a 315–435 keV energy window. Standards of the injected formulation were counted to determine the %ID g^−1^.

### Competition binding *in vivo* to identify the LSEC receptor

Mice (♂ and ♀, balb/c, Taconic) were placed into two groups of five mice. Each mouse in one group received an intraperitoneal injection of 0.90 ml of 20 g l^−1^ heparin (Sigma, >180 USP units per mg) in normal sterile saline (NSS) at time 0 and then 30 min later those mice received a IV bolus of 0.10 ml of 20 g l^−1^ heparin followed immediately by 0.01 mg of [^86^Y]fCNT in 0.10 ml via retroorbital sinus IV injection. The mice in the control group (*n*=5) received only a 0.01 mg dose of [^86^Y]fCNT in 0.10 ml via retroorbital sinus IV injection. At 1 h post injection of the radiolabeled fCNT, the mice were euthanized and the blood, liver, spleen and lymph nodes were harvested, weighed, and counted using a γ-counter.

### Immunofluorescence microscopy

Mice (♂, NCr/nu/nu) received 0.01 mg of fCNT in 0.10 ml via retroorbital sinus IV injection. Mice were euthanized at 1, 3, 5, 20, 40, 60, 180 min, 1, 3, 7 or 30 days and the liver, kidneys and spleen collected for IF analyses. Controls included naive tissue (no construct was injected) to determine baseline autofluorescence, hydrolyzed-AF488 dye that was not conjugated to SWCNT (at 1 and 60 min post injection), and isotype-control staining IgG (non-specific IgG as primary antibody). The immunofluorescent staining was performed in the Molecular Cytology Core Facility of Memorial Sloan-Kettering Cancer Center using Discovery XT processor (Ventana Medical Systems). Double and triple staining experiments were performed sequentially^55^. Specific details of the IF staining, microscopy and imaging are described in the Supplementary Information.

### Isolate LSEC and Kupffer cells from mouse liver

LSECs and Kupffer cells were extracted from mouse liver by collagenase perfusion (Liberase TM, Roche), via the portal vein^56^. LSEC were purified using anti-CD146 immunomagnetic beads (Miltenyi Biotec, Bergisch-Gladbach, Germany) and Kupffer cells similarly isolated using an anti-F4/80 biotin and streptavidin immunomagnetic beads (Miltenyi Biotec)^57^. The identity and purity of the two populations was analysed by flow cytometry, using Fluorescein isothiocyanate-labelled check reagents (Miltenyi Biotec), and by PCR analyses. Expression of the Kupffer cell marker *Emr1* and LSEC marker *Lyve1*, along with *Stab1* and *Stab2* was performed by RT-qPCR.

### Liver was examined for apoptosis and immune cell infiltration after fCNT

Murine liver sections (♀, balb/c, Taconic) were incubated in primary antibody solutions (rabbit anti-Alexa488 antibody (Molecular Probes) at 0.5 μg ml^−1^; rabbit anti-CD3 antibody (DAKO) at 1.2 μg ml^−1^; rabbit anti-Cleaved Caspase-3 antibody (Cell Signalling) at 0.1 μg ml^−1^; or TdT-biotin-dUTP (Roche) for TUNEL staining. Representative snapshots were taken from the scanned images and analysed for signal intensity or counts, normalized to tissue area, using image analysis software Metamorph (Molecular Devices, PA). Statistics (Student's *t*-tests) were performed and graphs were made using Prism6 (GraphPad, LaJolla, CA).

### Human hepatocyte microsphere toxicity study

Human liver microsphere tissue (Insphero, Glattbrugg, CH) was exposed to fCNT. Liver tissue spheres were exposed for 1, 2 or 3 days to 15 and 30 mg l^−1^ concentrations of fCNT *in vitro*. Hepatocyte microsphere tissue sections were stained with H&E, TUNEL (Promega, DeadEnd Colorimetric TUNEL System cat. no. G7130), and cleaved caspase-3 (Cell Signalling Technology, Cleaved Caspase-3 (Asp175) Antibody, cat. no. 9661).

### *In vitro* endocytosis assays

CHO cells were stably transfected with empty vector, full-length human Stabilin-1 and Stabilin-2 expression constructs were generated as described^31^,^58^,^59^. For endocytosis, fCNT labelled with AF488 were added in concentration 15 mg l^−1^ in serum free F12 medium for 30 min, 37 °C. AcLDL-AF488 (Life Technologies) in concentration 5 mg l^−1^ was used as a positive control for endocytic activity. Quantification of bound/internalized fluorescent ligands was performed with FACSCanto II flow cytometer (BD Biosciences) according to standard protocols. Data were visualized and analysed using FlowJo 7.6.5 software. For detection of fCNT endocytosis by confocal microscopy CHO cells were cultured on coverslips, fixed in PFA and stained using guinea pig anti-Stabilin-1 (clone GP2, self-produced) or mouse anti-Stabilin-2 (clone 3.1) abs as described^31^. fCNT were visualized using rabbit anti-AF488 antibody. Secondary antibodies were donkey anti-guinea pig or donkey anti-mouse labelled with Cy3, and donkey anti-rabbit-AF488 (all from Dianova). Confocal microscopy analysis was performed using Leica TCS SP8 microscope. Data were acquired and analysed with Leica Confocal software. CHO cells were pre-incubated with heparin (1 mg ml^−1^) in serum free F12 medium for 30 min 37 °C followed by addition of fCNT for another 30 min without changing medium. Endocytosed fCNT were detected by flow cytometry as described above.

### Data analyses

Three-dimensional region-of-interest analysis on PET images was performed with AsiPRO VM 5.0 software (Concorde Microsystems). Widefield and confocal microsopy images were evaluated using ImageJ (NIH, http://rsb.info.nih.gov/ij/), AxioVision LE (Zeiss), and Amira 4.1 (Visage Imaging, Inc.) software. Graphs were constructed and statistical data were evaluated using Graphpad Prism 3.0 (Graphpad Software, Inc.). Statistical comparison between two experimental groups was performed using a t-test (unpaired comparison).

### Data availability

The data that support the findings of this study are available from the corresponding author upon request.

## Additional information

**How to cite this article:** Alidori, S. *et al*. Deconvoluting hepatic processing of carbon nanotubes. *Nat. Commun.* 7:12343 doi: 10.1038/ncomms12343 (2016).

## Supplementary Material

Supplementary Information Supplementary Figures 1-17, Supplementary Methods and Supplementary References.
