# Supplementary material for: Deconvoluting hepatic processing of carbon nanotubes
Source: Nat Commun. 2016 Jul 29;7:12343. doi: 10.1038/ncomms12343 (PMC4974572; doi:10.1038/ncomms12343)
Supplement: Supplementary Information — Supplementary Figures 1-17, Supplementary Methods and Supplementary References. [file ncomms12343-s1.pdf]

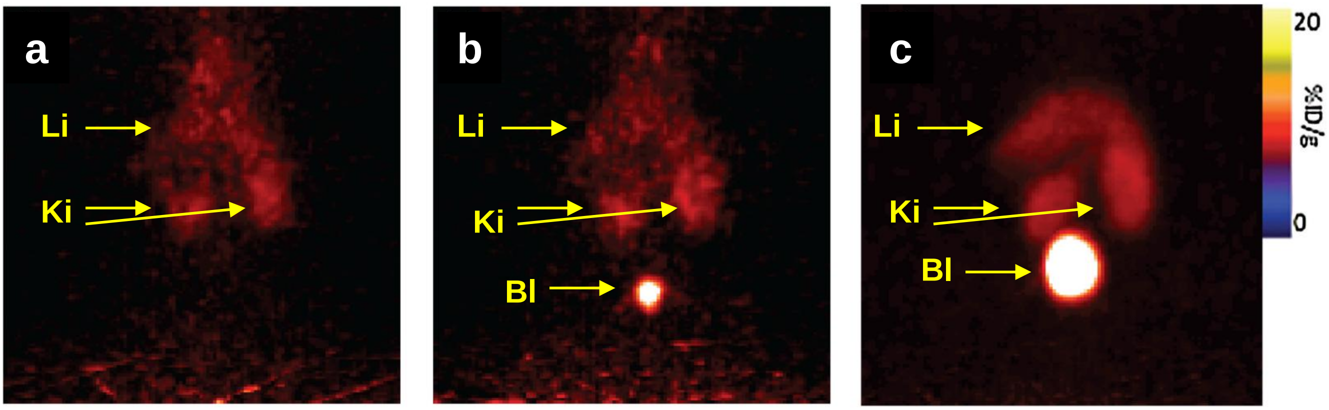

Supplementary Figure 1 PET projection images at (**a**) 3, (**b**) 20, and (**c**) 60 min post-injection of a representative mouse that received an intravenous injection of [^{86}Y]fCNT. The notations Ki, Li, and Bl indicate the kidneys, liver and bladder, respectively. The low image contrast in Panels **a** and **b** are a consequence of blood pool activity which is resolved by 60 minutes. n.b., The animal is positioned with head at the top and tail at the bottom of each panel image.

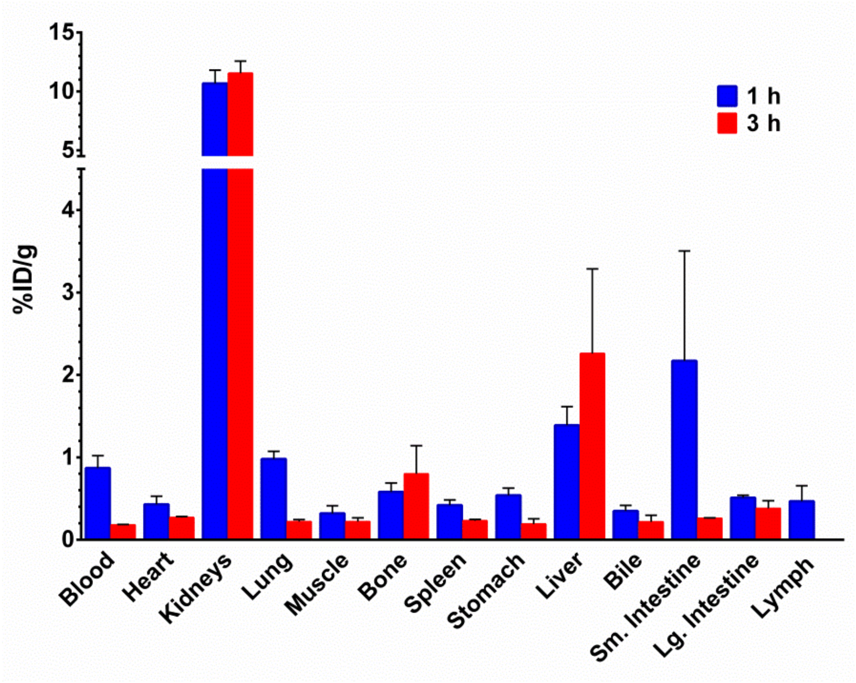

Supplementary Figure 2 Comprehensive [^{111}In]fCNT murine biodistribution data profile (%ID g^{-1} (mean ± SEM.) at 1h (blue bars) and 3h (red bars). There were 3-5 mice per group.

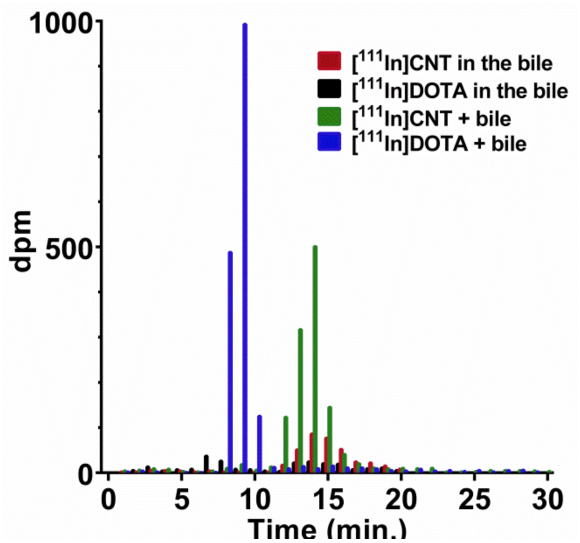

Supplementary Figure 3 Radiochromatographic analysis of bile harvested from mice injected with [^{111}In]fCNT showing intact hepatobiliary clearance of fCNT in vivo (red) versus an [^{111}In]DOTA-only control (black) in vivo. Note that very little activity from the [^{111}In]DOTA-only control cleared into the bile (the renal elimination route was preferred for these small molecules). Additional control traces include a physical mixture of [^{111}In]fCNT (green) and [^{111}In]DOTA-only (blue) with harvested bile (ex vivo) to ascertain the elution times of the non-injected materials mixed with bile.

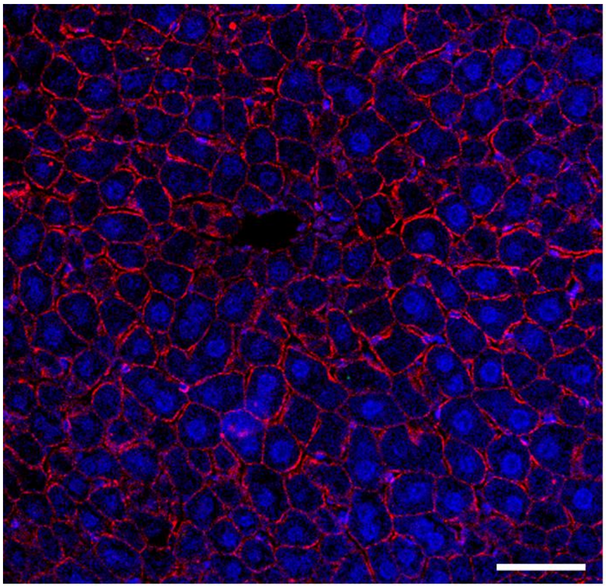

Supplementary Figure 4 Liver tissue from a control mouse that received only the AF488 fluorophore (not appended to fCNT) did not exhibit any AF488 signal in the liver. Anti-AF488 stain was applied to identify AF488; N-cadherin (red) was used to demarcate hepatocyte plasma membrane, and DAPI (blue) to mark nuclei (scale bar is 25 µm).

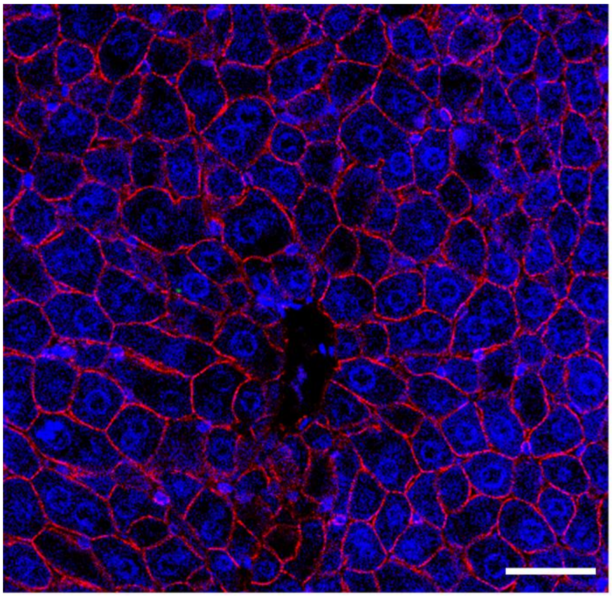

Supplementary Figure 5 Liver tissue from a control mouse that received only the injection vehicle did not exhibit any background staining in the liver. Anti-AF488 stain was applied, but there was no signal; N-cadherin (red) was used to demarcate hepatocyte plasma membrane, and DAPI (blue) to mark nuclei (scale bar is 25 μm).

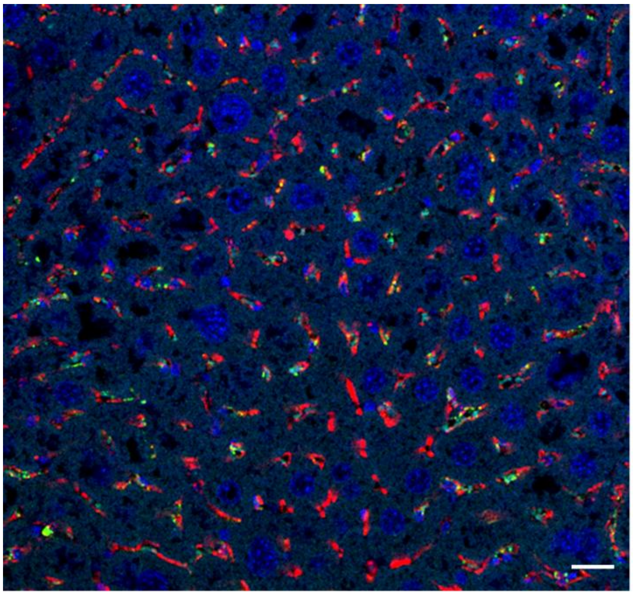

Supplementary Figure 6 Co-localization of fCNT and LSEC was seen in an image of liver at 1 hour. LSEC cells were marked with CD31 (red), nuclei with DAPI (blue) and fCNT with anti-AF488 (green) (scale bar is 25 μm).

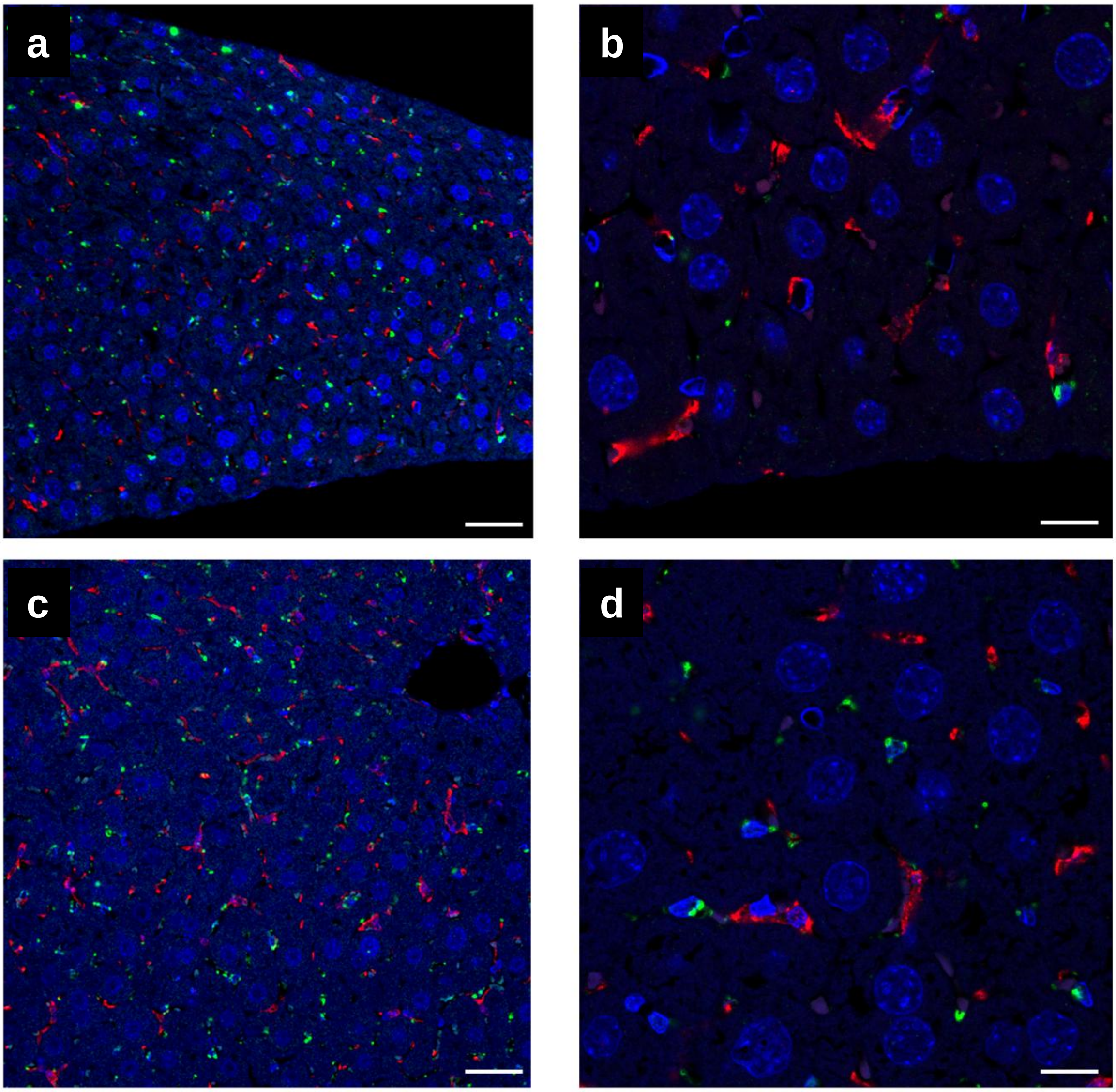

Supplementary Figure 7 Kupffer cells are resident-tissue macrophages that also populate the sinusoidal space but did not accumulate fCNT. (**a**) An image of liver at 1 hour showing Kupffer cells marked with the macrophage marker CSF-1R (red), nuclei with DAPI (blue), and fCNT with anti-AF488 (green) (scale bar is 25 μm). (**b**) A higher magnification of liver tissue stained for KC as in this figure panel **a**. The anti-AF488 signal was associated with adjacent small-nucleated cells in the sinusoidal space, but not any of the CSF-1R-marked cells (scale bar is 10 μm). (**c**) The absence of Kupffer cell co-localization with fCNT was confirmed using the Iba1 macrophage marker (Iba1 (red)/DAPI (blue)/anti-AF488 (green)) (scale bar is 25 μm). (**d**) A higher magnification of the tissue in this figure panel **c**, again showing the proximity of fCNT and macrophages, but not engulfment (scale bar is 10 μm).

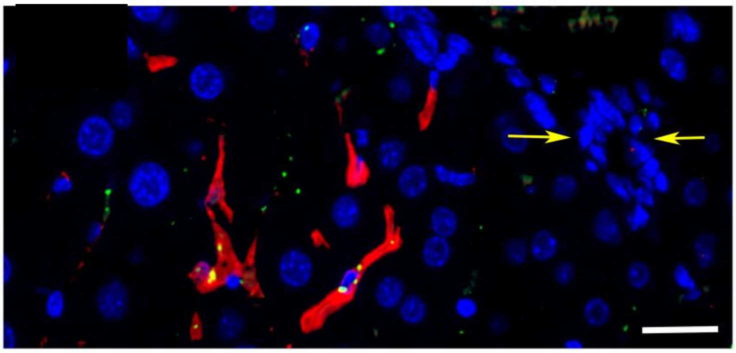

Supplementary Figure 8 The bile duct epithelium did not accrete fCNT (anti-AF488, green). The bile duct was identified morphologically using DAPI (blue) and the duct is shown by the yellow arrows; LSEC in frame were stained red with Lyve1 and did show fCNT clearance (scale bar is 25 μm).

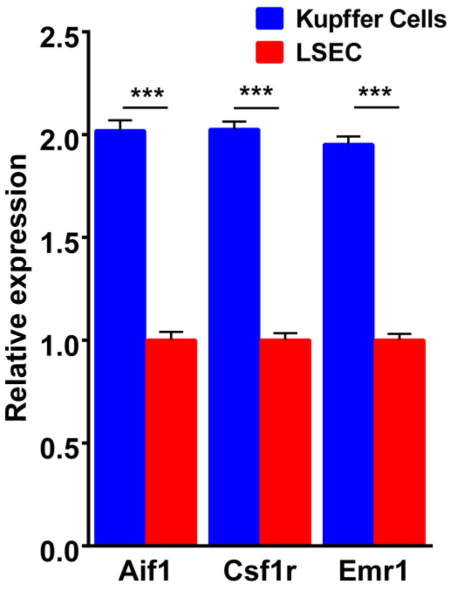

Supplementary Figure 9 PCR results of LSEC and Kupffer cells isolated from mouse liver confirmed the relative expression of *Emr1*, *Aif1*, and *Csfr1* mRNA by Kupffer cells versus LSEC.

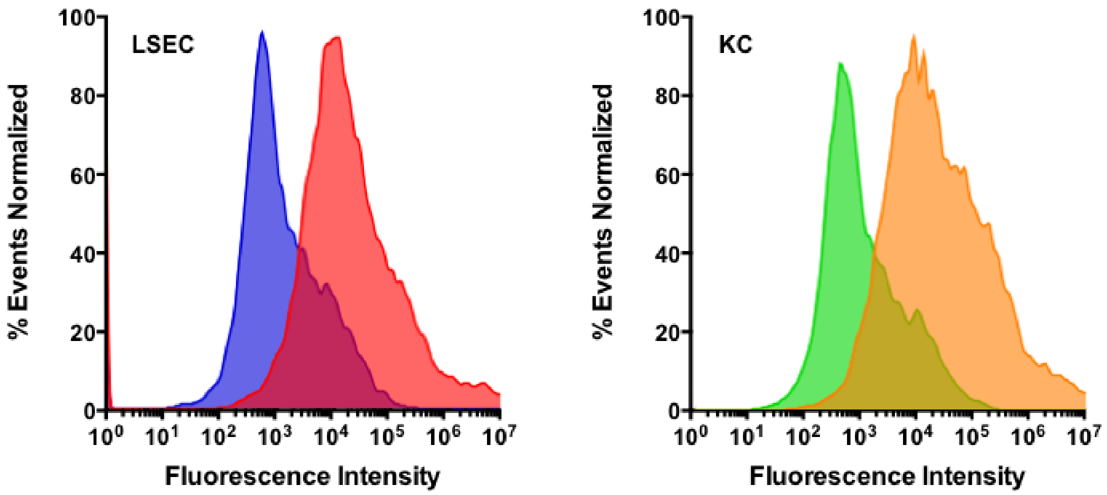

Supplementary Figure 10 FACS analysis was employed to confirm the identity of the cells isolated from mouse liver and used to perform PCR to measure relative Stabilin-1 and Stabilin-2 expression. The left panel shows purified LSEC stained with anti-CD146 immunomagnetic beads (red histogram) versus unstained LSEC cells (blue). The right panel shows the shift for isolated Kupffer cells stained with anti-F4/80 biotin and streptavidin immunomagnetic beads (orange histogram) versus unstained Kupffer cells (green histogram).

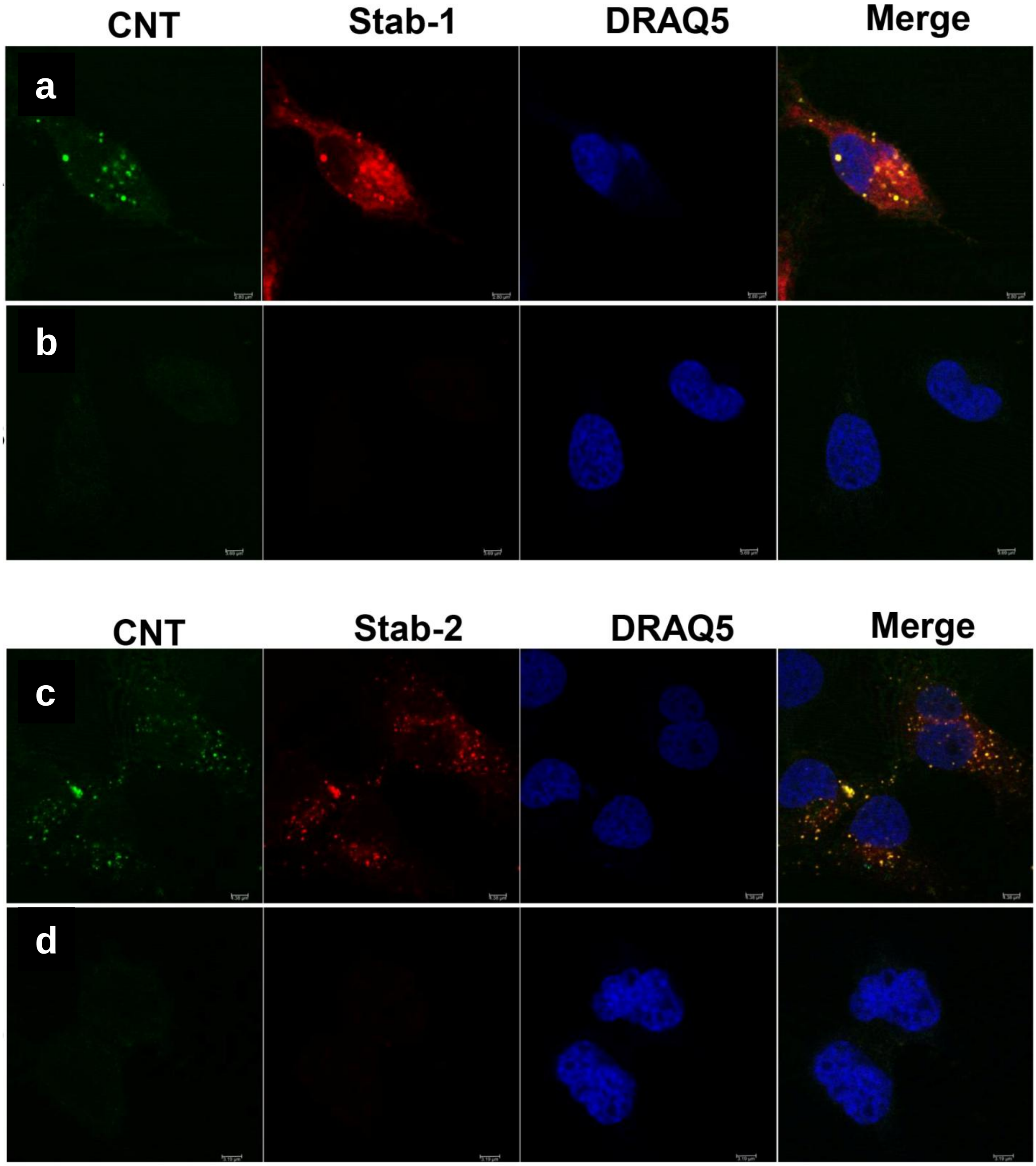

Supplementary Figure 11 Stabilin-1 and Stabilin-2 direct fCNT into an endocytic pathway. CHO cells transfected with Stabilin-1, Stabilin-2 or empty vector were exposed to fCNT for 30 min and then immunofluorescently stained for Stabilin-1 (red) and Stabilin-2 (red). Endocytosed fCNT (green) were visualized using rabbit anti-Alexa488 antibody as described in the experimental methods. fCNT clearance by Stabilin-1 and Stabilin-2 expressing clones is shown in the row with panels (**a**) and (**c**) respectively. fCNT clearance was not observed in empty vector-transfected cells (null control) and shown in the row with panels (**b**) and (**d**). Co-localization of stains for Stabilins (red) and fCNT (green) is shown in yellow punctate cellular bodies in the Merge columns. DRAQ5 was used to stain nuclei (blue). Scale bars are as follows: Panel (**a**) 2.80 µm; (**b**) 3.69 µm; (**c**) 4.38 µm; and (**d**) 3.19 µm.

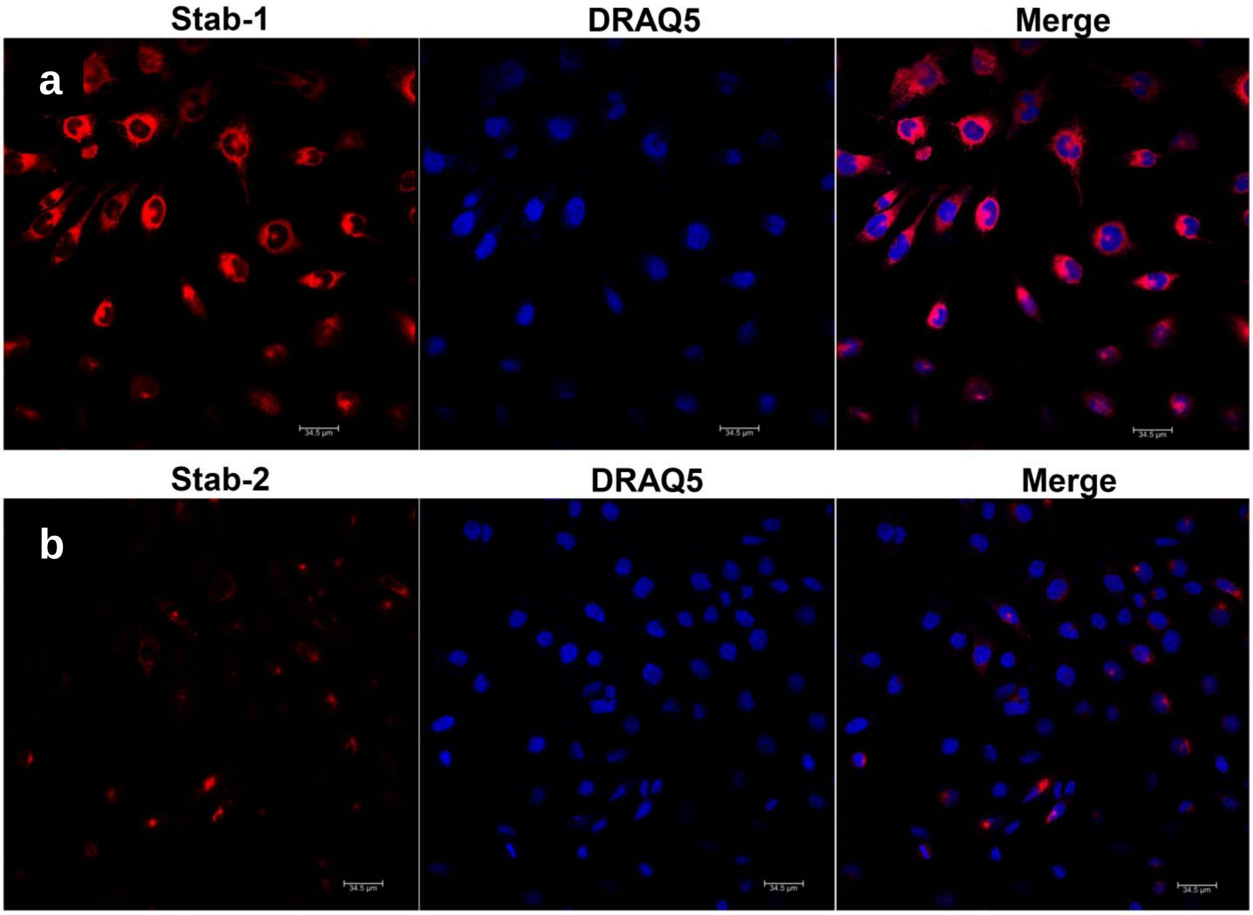

Supplementary Figure 12 The expression of Stabilin-1 and Stabilin-2 in stably transfected CHO cells is shown in (**a**) CHO-Stabilin-1 and (**b**) CHO-Stabilin-2 clones that were immunofluorescent stained red using anti-Stabilin-1 (clone GP2) or anti-Stabilin-2 (clone 3.1) antibodies and analyzed using confocal microscopy. DRAQ5 was used to stain nuclei (blue). Scale bars are 34.5 µM.

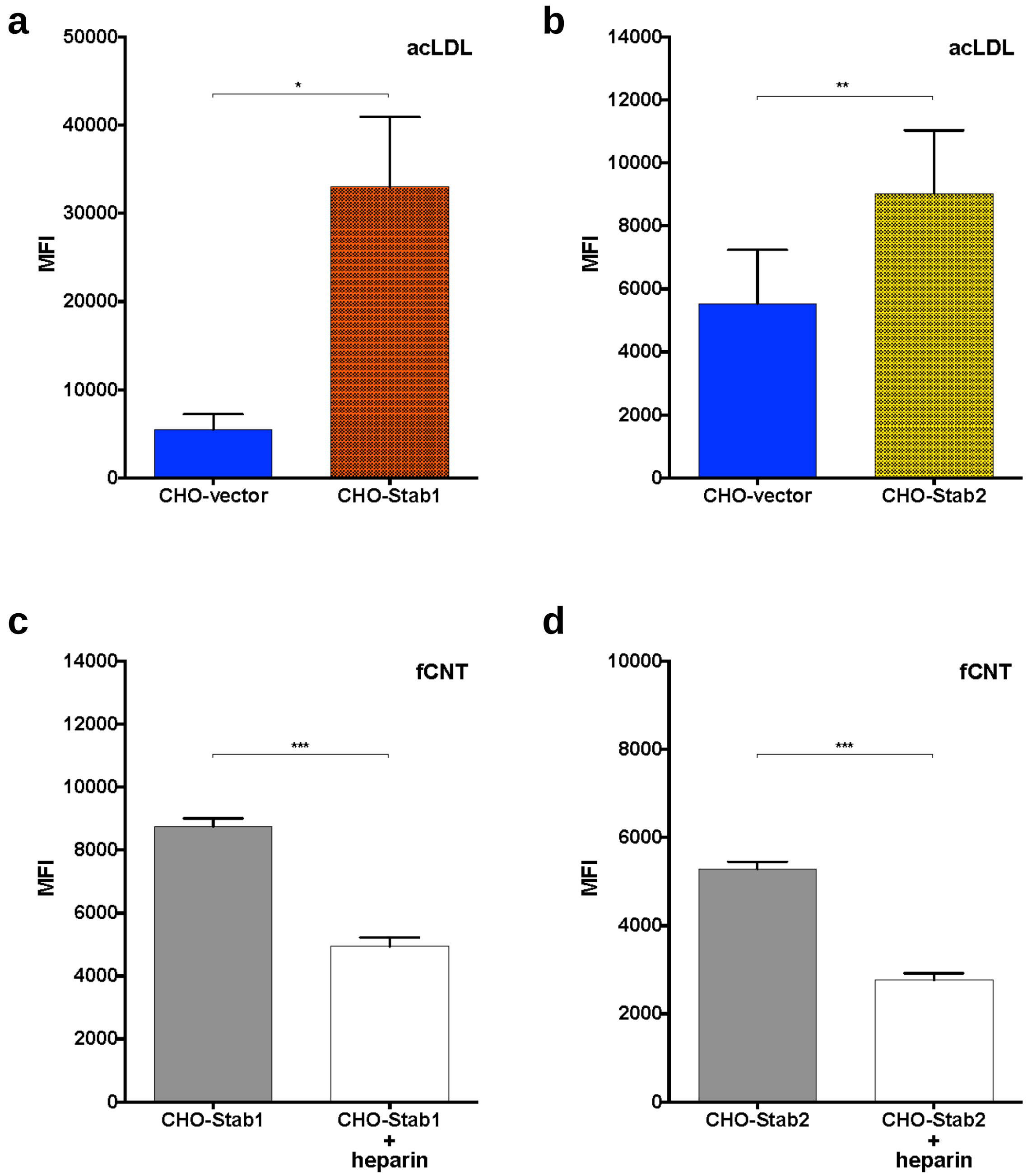

Supplementary Figure 13 Acetylated LDL (acLDL) clearance by Stabilin-1 and Stabilin-2 was examined as a positive endocytosis control. CHO cells stably transfected with empty vector, Stabilin-1 or Stabilin-2 expression constructs were incubated with acLDL-AF488 for 30 min followed by detection using rabbit anti-AF488 antibody by flow cytometry. Combined data from 4 experiments for acLDL binding are shown for (**a**) Stabilin-1 and (**b**) Stabilin-2 transfected CHO cells versus empty vector clones. Heparin competition for clearance of fCNT by these same (**c**) Stabilin-1 and (**d**) Stabilin-2 transfected CHO cells was also performed. CHO cells were pre-incubated with heparin (0.1 mg mL^{-1}) in serum free F12 medium for 30 min at 37^oC followed by addition of fCNT for another 30 min without changing media. Endocytosed fCNT were detected by flow cytometry as described above. The bars are mean ± SEM; *$P < 0.05$, **$P < 0.01$, ***$P < 0.001$, paired t-test.

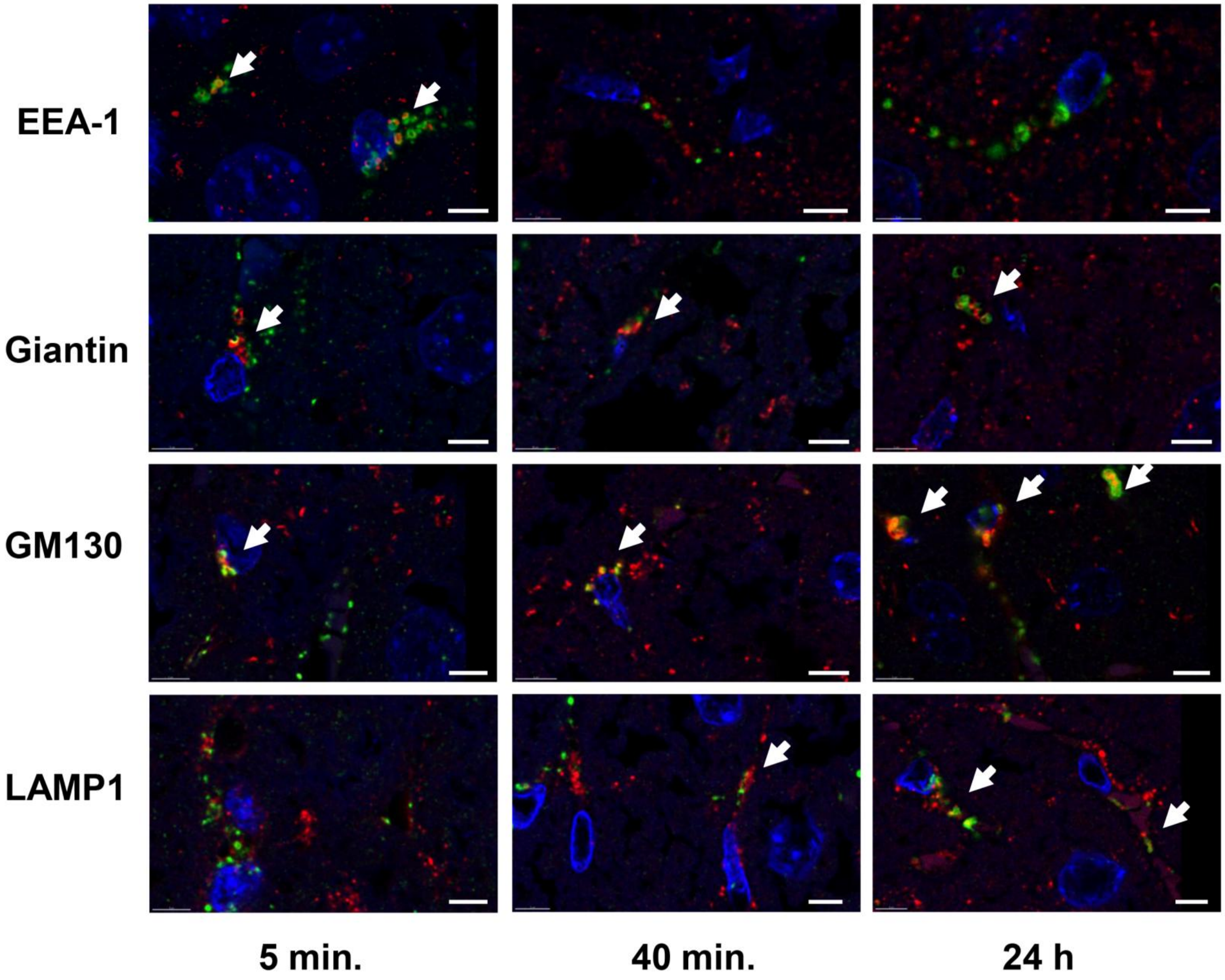

Supplementary Figure 14 LSEC internalized fCNT via Stabilin-mediated endocytosis and trafficked through the early endosome, Golgi apparatus and lysosome. Intracellular fCNT (anti-AF488 (green)) was observed in punctate patterns in mouse tissue and was shown to be associated with different intracellular organelles as a function of time. The kinetic traffic pattern is shown using a panel of 3D-rendered confocal images of the early endosome (EEA-1, red), Golgi compartment (Giantin (red) and GM130 (red)) and lysosome (LAMP1, red) as a function of time from administration. The nuclei are all stained with DAPI (blue) and scale bars are 5 μm). White arrows indicate co-localized AF488 signal with respective organelles. LAMP2 lysosome marker (data not shown) correlated with LAMP1 results.

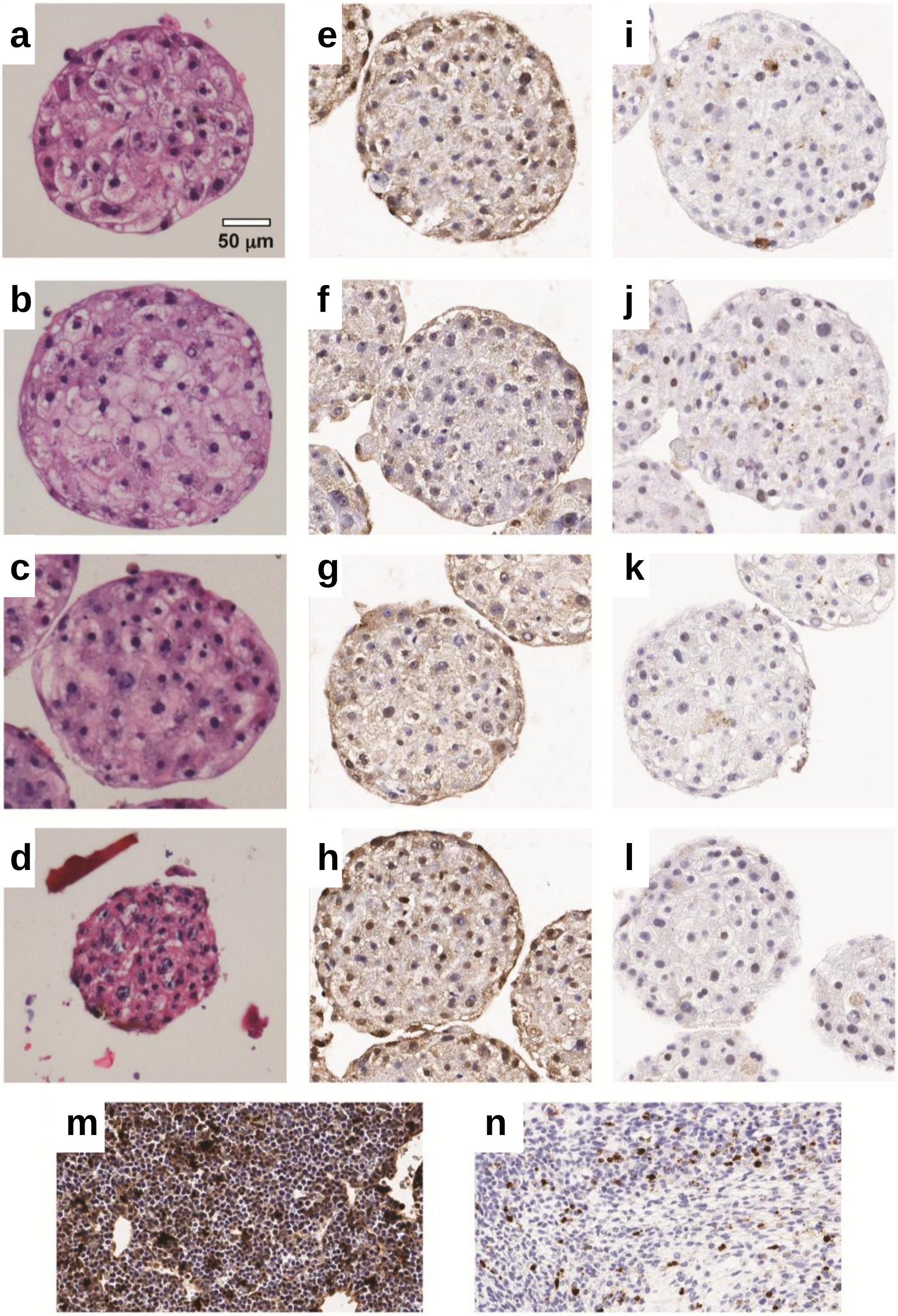

Supplementary Figure 15 Images of human hepatocyte microspheres exposed to 30 mg/L fCNT for 3 days in vitro remained viable. No morphological or apoptotic differences were observed by H&E (**a-d**), TUNEL (**e-h**) or cleaved caspase 3 (**i-l**) staining untreated control hepatocyte microsphere tissue versus hepatocyte microspheres treated for 1, 2, and 3 days, respectively. Liver and thoracic wall tissues from a C57BL/6J mouse hembrio were used as positive Tunel (**m**) and positive CC3 (**n**) staining controls, respectively. Scale bar is 50 µm in all images.

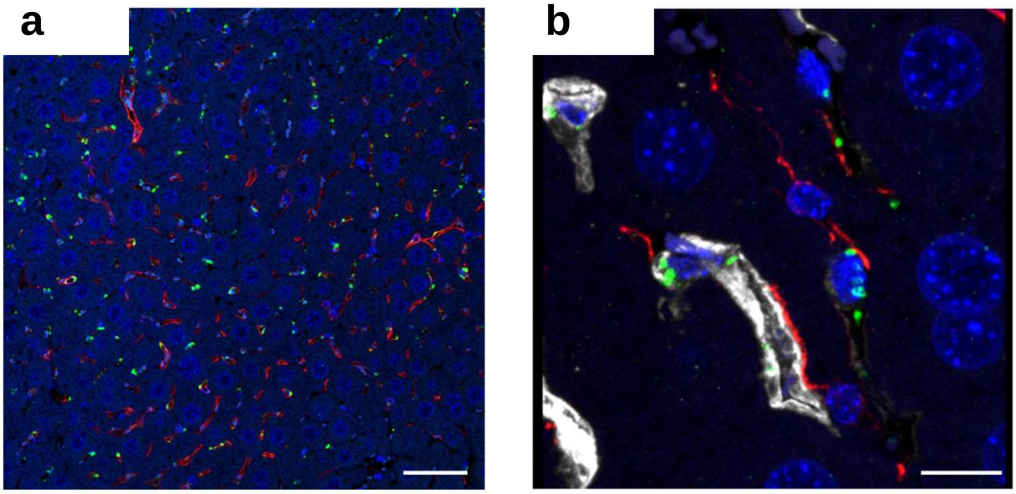

Supplementary Figure 16 Stellate cells also reside in the sinusoidal space. (**a**) An image of the liver at 1 hour showing stellate cells mapped with GFAP stain (red), nuclei with DAPI (blue), and fCNT with anti-AF488 (green) (scale bar is 25 µm). (**b**) Another liver tissue image of stellate cells marked with GFAP (red), nuclei with DAPI (blue), and fCNT with anti-AF488 (green) at higher magnification and with the addition of Lyve1 stain (white) to mark LSEC (scale bar is 10 µm). The LSEC and stellate cells are interleaved and in close proximity to each other.

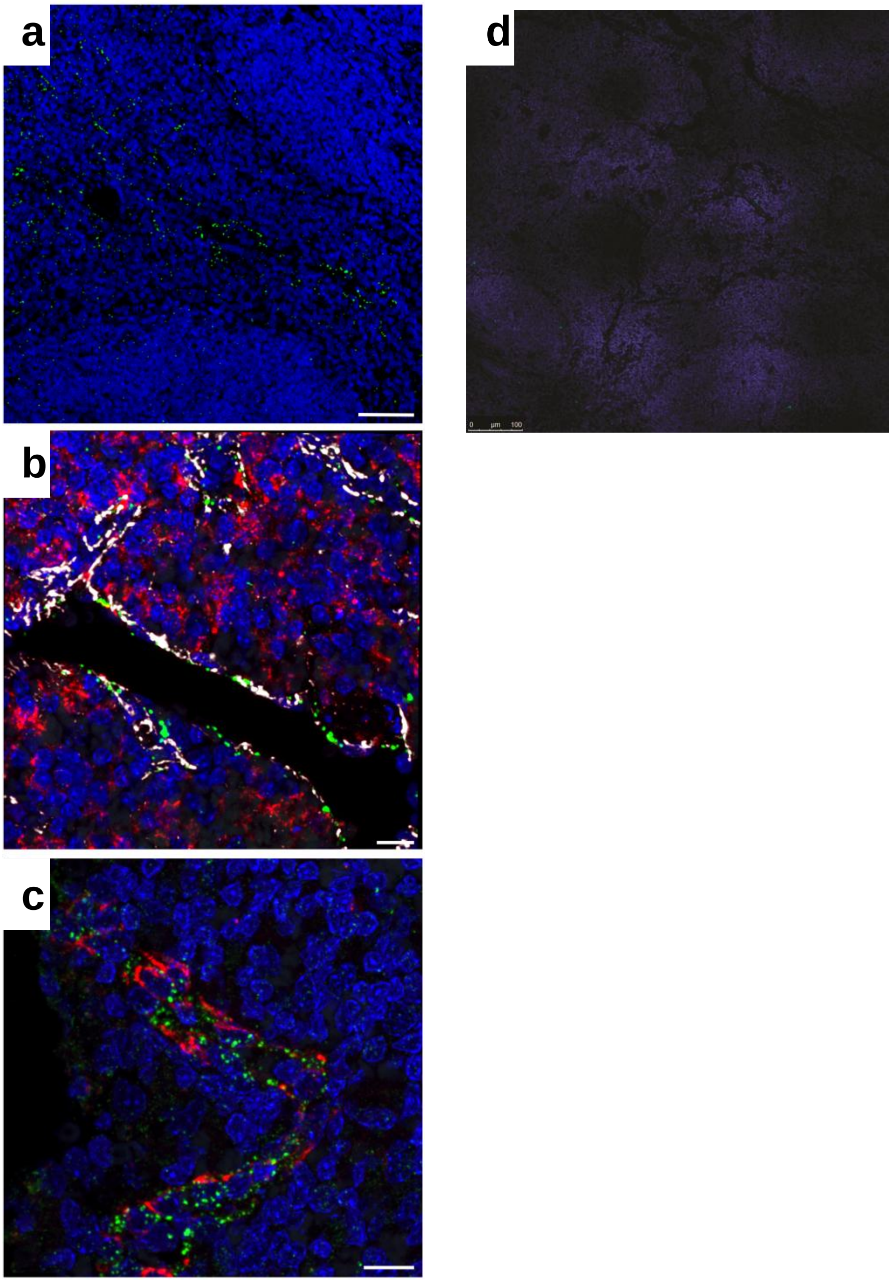

Supplementary Figure 17 Splenic sinusoidal endothelium and macrophages paralleled fCNT liver localization. (**a**) The fCNT (anti-AF488, green) also accumulated in the spleen, nuclei are DAPI stained (blue) (scale bar is 25 μm). (**b**) Images showing that fCNT accumulated in the splenic sinusoidal endothelial cells marked with β-catenin (white), but not splenic macrophages (Iba-1, red) at 1 hour post-administration. Nuclei were stained with DAPI (blue) and fCNT with anti-AF488 (green) (scale bar is 10 μm). (**c**) CD31 (red) confirmed that fCNT (anti-AF488, green) accumulated in spleen endothelial cells, nuclei were stained with DAPI (blue) (scale bar is 10 μm). **(d)** An image of a spleen section from a control mouse that was injected with only AF488 (no fCNT was appended to this small molecule dye) and no accumulation of dye was seen in this section stained for anti-AF488 (green) and nuclei (DAPI (blue) (scale bar is 100 μm).

Supplementary Methods

Synthesis and characterization of the soluble, functionalized single-walled carbon nanotube construct. HiPCO SWCNT (Unidym) were covalently functionalized with primary amines, Alexa Fluor 488 tetrafluorophenyl ester (AF488-TFP, Invitrogen), AF680-SE (Invitrogen), and 2-(4-isothiocyanatobenzyl)-1,4,7,10-tetraazacyclododecane-1,4,7,10-tetraacetic acid (DOTA, Macrocyclics) and purified as described (1). The amine loading per gram of SWCNT was determined using the Sarin assay as described previously (2). Transmission electron microscopy (TEM), dynamic light scattering (DLS), ζ-potential, chemical, chromatographic, spectroscopic, and Raman characterizations were performed as described previously (1,2) and confirmed the identity, stoichiometry and purity of the nanomaterial. The fCNT construct was analyzed by reverse phase high performance liquid chromatography on a Beckman Coulter System Gold chromatography system equipped with in-line UV/Vis spectrum detector and tunable multi-wavelength fluorescence detector (Jasco FP-2020). Radioactivity was monitored through the use of an inline γ-RAM Model 3 radioactivity detector (IN/US). The stationary phase was a Gemini (Phenomenex, Torrence, CA) C18 column column; the mobile phase was (A) 0.1M tetraethylammonium acetate (Aldrich), pH 6.5 and (B) acetonitrile at a flow rate of 1 mL min^{-1} using a 0 to 100 %B gradient over 30 minutes.

Radiolabeling with ^{86}Y and ^{111}In for imaging and biodistribution. The SWCNT-[([^{86}Y]DOTA)(AF488)(AF680)] construct was prepared by adding 300 MBq (8.1 mCi) of acidic ^{86}Y chloride (Memorial Sloan-Kettering Cancer Center Cyclotron Core and Washington University) to 0.400 mg of a 1 g L^{-1} solution of fCNT in metal-free water (MFW) and 0.050 mL of 3M ammonium acetate (Aldrich) and 0.015 mL of 150 g L^{-1} *l*-ascorbic acid (Aldrich) to yield a pH 5.0 solution. The solution was clear and dark green-brown in color. The reaction was heated at 60°C for 30 min, quenched with 0.040 mL of 50 mM diethylenetriaminepentaacetic acid (DTPA, Aldrich), and then purified by size exclusion chromatography using a P6 resin (BioRad) as the stationary phase and 1% human serum albumin (HSA, Swiss Red Cross) in 0.9% NaCl (Abbott Laboratories) as the mobile phase. An aliquot of the final product, [^{86}Y]fCNT, was used to determine the radiochemical purity by instant thin layer silica gel chromatography as described (2). Further spectroscopic, radiometric, and chromatographic characterization of the construct was performed by reverse phase HPLC as described (1,2). In-111 was obtained from MDS Nordion (Vancouver) for other tracer experiments. The SWCNT-[DOTA] constructs were labeled with ^{111}In using materials and methods similar to those described above for the ^{86}Y radiochemical labeling process. Both radionuclides have demonstrated similar labeling kinetics, purities, and yields in these labeling reactions (1-3).

Dynamic PET imaging to investigate pharmacokinetics. For all in vivo experiments, housing and care were in accordance with the Animal Welfare Act and the Guide for the Care and Use of Laboratory Animals. The animal protocols were approved by the Institutional Animal Care and Use Committee at MSKCC. Dynamic imaging was performed with the microPET Focus™ 120 (CTI Molecular Imaging) for the mouse model (♂, NCr/nu/nu, Taconic) as previously described (1). Dynamic PET/CT imaging was performed with a Siemens Biograph64 mCT PET/CT system for the non-human primate model (♂, *Macaca fascicularis*, Charles River). All PET images were acquired in time-of-flight mode commencing immediately after a 5 MBq intravenous (IV) injection of 5 mg [^{86}Y]fCNT. Acquisitions began with a 10×1 min dynamic sequence over the torso (heart, liver, kidney and bladder) and then transitioned to a series of whole body passes each consisting of 5 bed positions covering from the crown of the animal's head to mid-thigh.

The initial 6 passes were acquired at 1 min per bed, the next 6 at 2 min per bed and the final 6 at 3 min per bed, for a total imaging time (including gaps between scans) of about 195 minutes. Animals were maintained under 2% isoflurane/oxygen anesthesia during the scanning. The average decay-corrected activities in the hepatic volumes of interest (VOI) were converted to standardized uptake values (SUV) using the following calculation: SUV = activity concentration (kBq/mL)/(injection dose (MBq)/body weight (kg)). Time-activity curves (TAC) for dynamic analysis were generated using the uptake values for each VOI per frame. The SUV values were then plotted as a function of time to describe the kinetics. The parameters for quantitative ^{86}Y PET imaging were previously described (4).

The biodistribution and elimination was investigated in a naïve mouse model using tissue harvest. Mice (♂, NCr/nu/nu, Taconic) received an IV injection of [^{111}In]fCNT containing 0.04 mg of SWCNT construct and 74 kBq (0.002 mCi) of ^{111}In per mouse via the retroorbital sinus. The animals were placed into 4 groups of 3-5 mice per group. Each group was sacrificed with CO_2 aspiration at 1 h, 3 h, 24 h and 7d. Tissue samples (blood, heart, kidneys, muscle, bone, lung, stomach, liver, spleen, lymph nodes (superficial cervical, axillary, brachial, renal inguinal and lumbar), bile, small intestine (consisting of the duodenum, jejunum, and ileum), contents of the small intestine, large intestine (consisting of the cecum and colon), contents of the large intestine), and feces were harvested, weighed, and counted using a γ-counter (Packard Instrument Co.) with a 315 to 435 keV energy window. Standards of the injected formulation were counted to determine the %ID g^{-1}.

Competition binding in vivo to identify the liver sinusoidal endothelial cell receptor. Mice (♂ and ♀, balb/c, Taconic) were placed into two groups of 5 mice. Each mouse in one group received an intraperitoneal (IP) injection of 0.90 mL of 20 g L^{-1} heparin (Sigma) in normal sterile saline (NSS) at time 0 and then 30 min later those mice received a IV bolus of 0.10 mL of 20 g L^{-1} heparin followed immediately by 0.01 mg of [^{86}Y]fCNT in 0.10 mL via retroorbital sinus IV injection. The mice in the control group (n=5) received only a 0.01 mg dose of [^{86}Y]fCNT in 0.10 mL via retroorbital sinus IV injection. At 1 h post-injection of the radiolabeled fCNT, the mice were euthanized and the blood, liver, and spleens were harvested, weighed, and counted using a γ-counter. Standards of the injected formulation were counted to determine the %ID g^{-1}.

Immunofluorescence microscopy. Mice (♂, NCr/nu/nu) received 0.01 mg of fCNT in 0.10 mL via retroorbital sinus IV injection. Mice were euthanized at 1, 3, 5, 20, 40, 60, 180 min, 1d, 3d, 7d, or 30d and the liver, kidneys, and spleen harvested for IF analyses. Controls included naïve tissue (no construct was injected) to determine baseline autofluorescence, hydrolyzed-AF488 dye that was not conjugated to SWCNT (at 1 and 60 min post-injection), and isotype-control staining IgG (non-specific IgG as primary antibody). Harvested tissue was washed in ice cold PBS and fixed overnight in 4% paraformaldehyde at 4°C, embedded in paraffin, and sectioned to obtain 0.005 mm thick samples. Specific details of the IF staining are described below. Widefield microscopy was performed with an Axioplan2 imaging microscope, equipped with AxioCam MRm Camera (Zeiss, Inc), using filter cubes for DAPI, AF488 and TRITC. Slides were also scanned with the FLASH scanner (Perkin Elmer) to get an overview of the tissue. Confocal microscopy was performed using an Inverted Leica TCS SP5 microscope (Leica Microsystems, Inc). All 3D rendering has been done with Imaris (Bitplane).

Immunofluorescent staining protocols. The immunofluorescent staining was performed in the Molecular Cytology Core Facility of Memorial Sloan Kettering Cancer Center using Discovery XT processor (Ventana Medical Systems). Double and triple immunofluorescence experiments were performed sequentially (5). The tissue sections were deparaffinized with EZPrep buffer (Ventana Medical Systems), antigen retrieval was performed with CC1 buffer (Ventana Medical Systems) and sections were blocked for 30 minutes with Background Buster solution (Innovex) for anti-Alexa488, beta-catenin, Iba1, CSF-1R, GM130 and GFAP antibodies or with 10% normal rabbit serum (Vector Labs) in PBS for anti-CD31, Lyve1 and LAMP2 antibodies. Anti-AF488 (Molecular Probes, cat. no. A-11094, 5 µg mL^{-1}), anti-β-catenin (Sigma Aldrich, cat. no. C2206, 5 µg mL^{-1}), anti-Iba1 (Wako, cat. no. 019-19741, 0.5 µg mL^{-1}), anti-CSF-1R (Santa Cruz, cat. no. sc-692, 0.5 µg mL^{-1}), anti-EEA1 (Abcam, cat. no. ab2900, 0.8 µg mL^{-1}), anti-Giantin (Abcam, cat. no. ab24586, 1:400), and anti-GFAP (DAKO, cat. no. Z0334, 1 µg mL^{-1}) antibodies were applied and sections were incubated for 5 hours, followed by 60 min incubation with biotinylated goat anti-rabbit IgG (Vector labs, cat. no. PK6101, 1:200 dilution). Anti-CD31 (Dianova, cat. no. DIA-310, 1 µg mL^{-1}) and anti-LAMP2 (Abcam, cat. no. ab13524, 0.5 µg mL^{-1}) antibodies were applied and sections were incubated for 5 hours, followed by 60 min incubation with biotinylated rabbit anti-rat IgG (Vector labs, cat. no. PK-4004,1:200 dilution). Anti-Lyve1 (R&D Systems, cat. no. AF2125, 1 µg mL^{-1}) antibodies were applied and sections were incubated for 3 h, followed by 60 min incubation with biotinylated rabbit anti-goat IgG (Vector, cat. no. BA-5000,1:200 dilution). Anti-GM130 (BD Pharmingen, cat. no. 610823, 1 µg mL^{-1}) antibodies were applied and sections were incubated for 3 h, followed by 60 min incubation with biotinylated horse anti-mouse IgG (Vector Labs, cat. no. MKB-22258, 1:200 dilution). The detection was performed with Streptavidin-HRP D (DABMap kit, Ventana), followed by incubation with one of the following Tyramide Alexa Fluors (Invitrogen): AF488 (cat. no. T20922) only for anti-AF488, anti-AF546 (cat. no. T20933), anti-AF568 (cat. no. T20914), anti-AF594 (cat. no. T20935) or anti-AF647 (cat. no. T20936) prepared according to the manufacturer instructions with predetermined dilutions. Slides were counterstained with DAPI (Sigma Aldrich, cat. no. D9542, 5 µg mL^{-1}) for 10 min and coverslipped with Mowiol.

Isolate LSEC and Kupffer cells from mouse liver. Liver sinusoidal endothelial cells and Kupffer cells were extracted from mouse liver by collagenase perfusion (Liberase TM, Roche), via the portal vein (6). The liver was removed and the tissue mechanically disrupted to release cells. Hepatocytes were excluded by low speed (30×g) centrifugation from the cell mixture. The resulting nonparenchymal cell suspension, containing Kupffer and LSEC, was pelleted at high speed (350×g) centrifugation for 10 minutes, washed in 0.1% BSA-enriched PBS, Fc receptor blocked and split into two fractions (approximately 3E8 cells each). LSEC were purified using anti-CD146 immunomagnetic beads (Miltenyi Biotec, Bergisch-Gladbach, Germany) and Kupffer cells similarly isolated using an anti-F4/80 biotin and streptavidin immunomagnetic beads (Miltenyi Biotec) (7). The isolation and purification process yielded about 2E6 cells per population. The identity and purity of the two populations was analyzed by flow cytometry, using FITC labeled check reagents (Miltenyi Biotec), and by PCR analyses.

Gene expression for LSEC markers. Expression of the Kupffer cell marker *Emr1* and LSEC marker *Lyve1,* along with *Stab1* and *Stab2* was performed by RT-qPCR. Cells were isolated as described and RNA was extracted from using Qiagen RNAEasy Minikit (#74106). RNA was converted into cDNA using qScript™ cDNA SuperMix (Quanta Biosciences Gaithersburg, MD

USA). Primers for mouse *Aif1* (Mm00479862_g1), *CSF1R* (Mm01266652_m1), *Emr1* (Mm00802529_m1), *Lyve1* (Mm00475056_m1), *Stab1* (Mm00460390_m1), and *Stab2* (Mm00454684_m1) were obtained from Applied Biosystems. For assessment of gene expression using RT-PCR PerfeCTa® FastMix® II (Quanta), reactions were carried out in triplicates using 1.6 ng cDNA and standard thermocycling conditions (2 minutes at 50ºC, 10 minutes at 95ºC, 40 cycles of 15 seconds at 95ºC, and 1 minute at 60ºC). Data were normalized to *R18s* (Mm04277571).

TUNEL, cleaved caspase 3, and CD3 staining of mouse tissue to examine apoptosis and inflammation. All samples were fixed in freshly prepared 4% paraformaldehyde in PBS (Electron Microscopy Sciences) for 16-24 hours at 4°C. They were washed in PBS and kept in 70% ethanol for up to several days at 4°C. Tissues were processed for paraffin embedding using Leica ASP6025 tissue processor and embedded in paraffin (ParaPlast Plus, Leica Microsystems). Paraffin blocks were soaked in ice-water mixture for 30 min, then an automated paraffin microtome (RM2265, Leica Microsystems, Germany) was used to obtain 5 µm thick sections. The sections were mounted onto SuperfrostPlus microscope slides (Fisher Scientific) and stored at 4°C until ready for staining. Slides were loaded onto the autostainer Discovery XT processor (Ventana Medical Systems) and de-waxed by being heated up to 98°C with EZPrep solution (Ventana Medical Systems). Antigen retrieval was performed with CC1 buffer (Ventana Medical Systems) before subsequent steps. Slides were incubated in primary antibody solutions (rabbit anti-Alexa488 antibody (Molecular Probes) at 0.5 µg mL^{-1}; rabbit anti-CD3 antibody (DAKO) at 1.2 µg mL^{-1}; rabbit anti-Cleaved Caspase 3 antibody (Cell Signaling) at 0.1 µg mL^{-1}; or TdT-biotin-dUTP (Roche) for TUNEL staining. The biotinylated secondary antibody was applied for 60 min. Fluorescent detection was performed with Blocker D, Streptavidin-HRP D (Ventana Tyramid-AlexaFluor of various wavelengths (Invitrogen). Slides were stained with 5µg mL^{-1} of 4',6-diamidino-2-phenylindole (DAPI, Sigma Aldrich), and mounted with Mowiol 4-88 mounting media (Calbiochem) and #1.5 cover glass. Slides were stored for minimum overnight at -20°C before imaging. The slides were equilibrated to room temperature and digitally scanned using Pannoramic Flash (3DHistec, Hungary) with 20×/0.8NA objective. Representative snapshots were taken from the scanned images and analyzed for signal intensity or counts, normalized to tissue area, using image analysis software Metamorph (Molecular Devices, PA). Statistics (student T-tests) were performed and graphs were made using Prism6 (GraphPad, LaJolla, CA).

Human hepatocyte microsphere toxicity study. Human liver microsphere tissue (Insphero, Glattbrugg, CH) was exposed to fCNT. Liver tissue spheres were exposed for 1, 2, or 3 days to 15 and 30 mg L^{-1} concentrations of fCNT in vitro. Hepatocyte microsphere staining with hematoxylin and eosin (H&E), TUNEL (Promega, DeadEnd™ Colorimetric TUNEL System cat. no. G7130), and cleaved caspase 3 (Cell Signalling Technology, Cleaved Caspase-3 (Asp175) Antibody, cat. no. 9661) was used to investigate morphological, apoptotic, and DNA damage differences between treated and vehicle treated control spheres over the 72 h exposure time course. Liver and thoracic wall tissues from a C57BL/6J mouse hembrio were used as positive Tunel and positive CC3 staining controls, respectively.

In vitro endocytosis assays. CHO cells stably transfected with empty vector, full-length human Stabilin-1 and Stabilin-2 expression constructs were generated as described (8-10) and

propagated in F12 Nut Mix (1X) + Glutamax medium (Gibco) supplemented with 10% fetal bovine serum, 1% penicillin/streptomycin (both from Biochrom) and 700 µg mL^{-1} G418 (Merck Millipore). For endocytosis, fCNT labeled with AF488 were added in concentration 15 µg mL^{-1} in serum free F12 medium for 30 min, 37°C. AcLDL-AF488 (Life Technologies) in concentration 5 µg mL^{-1} was used as a positive control for endocytic activity. Cells were harvested by trypsinization and fixed in 2% PFA for 10 min, RT. Endocytosed fCNT were detected by intracellular staining using rabbit anti-AF488 antibodies (Life Technologies) followed by staining with $F(ab')_2$ fragment donkey anti-rabbit APC-conjugated abs (Jackson Immunoresearch Laboratories). Quantification of bound/internalized fluorescent ligands was performed with FACSCanto II flow cytometer (BD Biosciences) according to standard protocols. Data were visualized and analyzed using FlowJo 7.6.5 software. For detection of fCNT endocytosis by confocal microscopy CHO cells were cultured on coverslips, fixed in PFA and stained using guinea pig anti-Stabilin-1 (clone GP2, self-produced) or mouse anti-Stabilin-2 (clone 3.1) abs as described (9). fCNT were visualized using rabbit anti-AF488 antibody. Secondary antibodies were donkey anti-guinea pig or donkey anti-mouse labeled with Cy3, and donkey anti-rabbit-AF488 (all from Dianova). Confocal microscopy analysis was performed using Leica TCS SP8 microscope. Data were acquired and analyzed with Leica Confocal software. Heparin competition for fCNT clearance was performed with the Stabilin-1 and Stabilin-2 transfected CHO cells pre-incubated with heparin (1 mg mL^{-1}) in serum free F12 medium for 30 min at 37°C followed by addition of fCNT for another 30 min without changing media. Endocytosed fCNT were detected by flow cytometry as described above.

Data Analyses. Three-dimensional region-of-interest analysis on PET images was performed with AsiPRO VM 5.0 software (Concorde Microsystems). Widefield and confocal microsopy images were evaluated using ImageJ (NIH, http://rsb.info.nih.gov/ij/), AxioVision LE (Zeiss), and Amira 4.1 (Visage Imaging, Inc.) software. Graphs were constructed and statistical data were evaluated using Graphpad Prism 3.0 (Graphpad Software, Inc.). Statistical comparison between 2 experimental groups was performed using a t-test (unpaired comparison).

Supplementary References

1. Ruggiero A*, et al.* Paradoxical glomerular filtration of carbon nanotubes. *P Natl Acad Sci USA* **107**, 12369-12374 (2010).
2. McDevitt MR*, et al.* Tumor targeting with antibody-functionalized, radiolabeled carbon nanotubes. *J Nucl Med* **48**, 1180-1189 (2007).
3. Ruggiero A*, et al.* Imaging and treating tumor vasculature with targeted radiolabeled carbon nanotubes. *Int J Nanomed* **5**, 783-802 (2010).
4. Beattie BJ, Finn RD, Rowland DJ, Pentlow KS. Quantitative imaging of bromine-76 and yttrium-86 with PET: a method for the removal of spurious activity introduced by cascade gamma rays. *Medical physics* **30**, 2410-2423 (2003).
5. Yarilin D*, et al.* Machine-based method for multiplex in situ molecular characterization of tissues by immunofluorescence detection. *Sci Rep-Uk* **5**, (2015).
6. Smedsrød B. Protocol for preparation of mouse liver Kupffer cells and liver sinusoidal endothelial cells., (2012).
7. Liu W*, et al.* Sample preparation method for isolation of single-cell types from mouse liver for proteomic studies. *Proteomics* **11**, 3556-3564 (2011).

8. Kzhyshkowska J, Gratchev A, Brundiers H, Mamidi S, Krusell L, Goerdt S. Phosphatidylinositide 3-kinase activity is required for stabilin-1-mediated endosomal transport of acLDL. *Immunobiology* **210**, 161-173 (2005).
9. Schledzewski K*, et al.* Deficiency of liver sinusoidal scavenger receptors stabilin-1 and-2 in mice causes glomerulofibrotic nephropathy via impaired hepatic clearance of noxious blood factors. *J Clin Invest* **121**, 703-714 (2011).
10. Kzhyshkowska J. Multifunctional Receptor Stabilin-1 in Homeostasis and Disease. *Thescientificworldjo* **10**, 2039-2053 (2010).
